# *Synechocystis* sp. PCC 6803 Requires the Bidirectional Hydrogenase to Metabolize Glucose and Arginine Under Oxic Conditions

**DOI:** 10.3389/fmicb.2022.896190

**Published:** 2022-05-31

**Authors:** Heinrich Burgstaller, Yingying Wang, Johanna Caliebe, Vanessa Hueren, Jens Appel, Marko Boehm, Sinje Leitzke, Marius Theune, Paul W. King, Kirstin Gutekunst

**Affiliations:** ^1^Plant Cell Physiology and Biotechnology, Botanical Institute, University of Kiel, Kiel, Germany; ^2^Molecular Plant Physiology, Bioenergetics in Photoautotrophs, University of Kassel, Kassel, Germany; ^3^National Renewable Energy Laboratory, Biosciences Center, Golden, CO, United States

**Keywords:** hydrogenase, diaphorase, photosynthetic complex I (NDH-1), photosynthesis, respiration, arginine, photomixotrophy

## Abstract

The cyanobacterium *Synechocystis* sp.PCC 6803 possesses a bidirectional NiFe-hydrogenase, HoxEFUYH. It functions to produce hydrogen under dark, fermentative conditions and photoproduces hydrogen when dark-adapted cells are illuminated. Unexpectedly, we found that the deletion of the large subunit of the hydrogenase (HoxH) in *Synechocystis* leads to an inability to grow on arginine and glucose under continuous light in the presence of oxygen. This is surprising, as the hydrogenase is an oxygen-sensitive enzyme. In wild-type (WT) cells, thylakoid membranes largely disappeared, cyanophycin accumulated, and the plastoquinone (PQ) pool was highly reduced, whereas Δ*hoxH* cells entered a dormant-like state and neither consumed glucose nor arginine at comparable rates to the WT. Hydrogen production was not traceable in the WT under these conditions. We tested and could show that the hydrogenase does not work as an oxidase on arginine and glucose but has an impact on the redox states of photosynthetic complexes in the presence of oxygen. It acts as an electron valve as an immediate response to the supply of arginine and glucose but supports the input of electrons from arginine and glucose oxidation into the photosynthetic electron chain in the long run, possibly *via* the NDH-1 complex. Despite the data presented in this study, the latter scenario requires further proof. The exact role of the hydrogenase in the presence of arginine and glucose remains unresolved. In addition, a unique feature of the hydrogenase is its ability to shift electrons between NAD(H), NADP(H), ferredoxin, and flavodoxin, which was recently shown *in vitro* and might be required for fine-tuning. Taken together, our data show that *Synechocystis* depends on the hydrogenase to metabolize organic carbon and nitrogen in the presence of oxygen, which might be an explanation for its prevalence in aerobic cyanobacteria.

## Introduction

The bidirectional NiFe-hydrogenase of the cyanobacterium *Synechocystis* is composed of a diaphorase sub-complex (HoxEFU), which reacts with its redox partners NAD(P)(H), ferredoxin, and flavodoxin and a hydrogenase sub-complex (HoxYH), which catalyzes the production and consumption of hydrogen (H_2_) (Gutekunst et al., 2014; Artz et al., 2020). HoxEFUYH is the only hydrogenase encoded in this organism. It is localized in the cytoplasm and is found to associate dynamically with thylakoid membranes (Burroughs et al., 2014). The production of H_2_ occurs under fermentative growth conditions as a means for balancing redox poise. Furthermore, at the onset of photosynthesis in dark-adapted cells, HoxEFUYH catalyzes hydrogen production (photohydrogen) during the brief anaerobic phase of growth. Under these conditions, surplus electrons from the photosynthetic electron chain that are not accepted by the Calvin–Benson–Bassham (CBB) cycle are transferred to the hydrogenase and utilized for H_2_ production. In this situation, the enzyme works as an electron valve to protect the photosynthetic electron chain from over reduction (Appel et al., 2000; Cournac et al., 2004). Photohydrogen is subsequently oxidized by HoxEFUYH, and the electrons are most likely transferred to the photosynthetic electron chain *via* PQ (Dutta and Vermaas, 2016). As soon as oxygen accumulates due to H_2_O splitting at photosystem II (PSII), hydrogen is neither produced nor oxidized. The current model assumes that exposure to oxygen blocks the active site of the NiFe-hydrogenase *via* a hydroxy (OH^−^) group that bridges Ni and Fe in the active site of HoxH and thereby prevents H_2_-turnover (Pandelia et al., 2010; McIntosh et al., 2011; Caserta et al., 2020). Due to its susceptibility to inactivation, the enzyme is considered oxygen sensitive. Therefore, it is remarkable that the enzyme is widespread in cyanobacteria, which seldomly encounter anoxic conditions in their natural environment. It is also worthy to note that the enzyme is constitutively expressed in *Synechocystis* under standard laboratory conditions, i.e., continuous light and constant O_2_ supersaturation. Distinct subcomplexes of the enzyme (HoxEFU, HoxFUYH, and HoxFU) have been detected in addition to the complete HoxEFUYH pentameric enzyme *in vivo* (Eckert et al., 2012). However, it is still unknown whether these subcomplexes fulfill specific functions.

*Synechocystis* can grow on a variety of different nitrogen sources. For example, cells can consume either inorganic nitrogen in the form of nitrate and ammonium or organic nitrogen such as urea or arginine and glutamine (Flores and Herrero, 1994). Nitrate is reduced intracellularly to nitrite and ammonium, which is subsequently incorporated into biomass (Forchhammer and Selim, 2020). It has previously been shown that the hydrogenase is essential for growth in a dark-light cycle under mixotrophic conditions when nitrate is replaced by arginine as the nitrogen source (Gutekunst et al., 2014). The replacement of nitrate with arginine has a dramatic effect on the metabolism and phenotype of *Synechocystis*. Arginine can readily be taken up by the cells and metabolized as a cellular nitrogen source and in the production of cyanophycin (Stephan et al., 2000; Schriek et al., 2007). Cyanophycin consists of equimolar amounts of aspartic acid and arginine and can be quickly metabolized when required. Besides the accumulation of cyanophycin, cells cultivated on arginine also disassemble and restructure the thylakoid membranes, have reduced photosynthetic activity, and appear yellow due to a reduced chlorophyll content (Stephan et al., 2000). The addition of nitrate in combination with arginine partly rescues this phenotype (Stephan et al., 2000). The Δ*psbO* mutant has a compromised water-splitting apparatus at PSII and displays reduced H_2_O oxidizing capacities. It grows better than the wild-type (WT) on arginine, stays green, and displays only minor morphological changes (Stephan et al., 2000). The difference between the WT and the Δ*psbO* mutant strain is even more pronounced at higher light intensities. While nitrate needs to be reduced by ferredoxin and is an important electron sink of the photosynthetic electron chain, arginine needs to be oxidized to be utilized for biosynthetic reactions. *Synechocystis* encodes enzymes for two distinct arginine oxidation pathways: the arginine deiminase pathway and the arginine dehydrogenase pathway (Schriek et al., 2007, 2009). The arginine deiminase pathway yields ${\text{NH}}_{4}^{+}$, NADH, and ATP, whereas the arginine dehydrogenase pathway yields ${\text{NH}}_{4}^{+}$, NAD(P)H, and succinate. The arginine deiminase in *Synechocystis* contains predicted transmembrane helices which implies that the enzyme could be membrane attached (Schriek et al., 2007). Arginine dehydrogenase has been clearly demonstrated to attach to the thylakoid membrane (Schriek et al., 2009). Electrons from arginine oxidation can either be fed directly into the plastoquinone (PQ)-pool *via* the arginine dehydrogenase or indirectly *via* succinate and the succinate dehydrogenase, which likewise transfers electrons into the PQ-pool (refer to photosynthetic electron transfer chain of Figure 8) (Schriek et al., 2009; Mullineaux, 2014). In contrast to nitrate, which accepts electrons from the photosynthetic electron chain, arginine reduces the PQ-pool. However, nitrate has the potential to relieve surplus electrons from photosynthesis, and arginine has the potential to overload the PQ pool and cause oxidative stress. This could be an explanation for the observation that the Δ*psbO* mutant with reduced H_2_0 splitting capacities is superior to the WT in metabolizing arginine especially in high light (Stephan et al., 2000; Schriek et al., 2009). The addition of glucose to cells cultured on arginine should enhance the supply of electrons by the activity of respiratory dehydrogenases that reduce the PQ pool (refer to photosynthetic electron transfer chain of Figure 8) (Lea-Smith et al., 2016; Wang et al., 2022). Photomixotrophic conditions on arginine should, therefore, lead to highly reducing conditions in the cells. The observation that Δ*hoxH* strain is unable to grow on arginine under mixotrophic dark-light conditions was interpreted as HoxEFUYH being required for electron dissipation *via* hydrogen production (Gutekunst et al., 2014). This interpretation appeared most obvious in cells during dark, fermentative H_2_ production, or during transient hydrogen production at the onset of illumination (photohydrogen production). However, surprisingly, we found in this study that the Δ*hoxH* strain was unable to grow under photomixotrophic conditions on arginine, even under continuous illumination and O_2_ saturating conditions. This is especially remarkable, as HoxH is known to be inactived in the presence of oxygen (McIntosh et al., 2011). To address this conundrum, investigations were undertaken to ensure the growth phenotype arose from the absence of HoxH and to elucidate the physiological function of the enzyme under these growth conditions. Our results provide strong evidence that under oxic conditions, the hydrogenase has a physiological function beyond hydrogen cycling and is required for photomixotrophic growth on organic carbon and nitrogen.

## Materials and Methods

### Construction of Mutants and Utilized Strains

All mutants that were constructed or utilized in this study are listed in Supplementary Table 1. All the primers used in this study are listed in Supplementary Table 2. All mutants were constructed in the non-motile GT WT of *Synechocystis* sp. PCC 6803 (Trautmann et al., 2012). Constructs for the deletion of the genes were generated by Gibson cloning (Gibson et al., 2009) assembling three fragments into the pBluescript SK(+) in a single step. After examination by sequencing, the plasmids were transformed into *Synechocystis* sp. PCC 6803 cells as described (Williams, 1988). In short, genes were replaced with antibiotic resistance cassettes *via* homologous recombination. The transformation efficiency is especially high in the exponential growth phase. Therefore, 250 ml *Synechocystis* cultures were inoculated in glass tubes (diameter of 3.5 cm) from a preculture with an OD_750_ of 0,15 on the day prior to transformation. On the day of transformation, the cells were harvested and resuspended in 600 μl BG11. A volume of 300 μl of the cell suspension was mixed with 6–18 μg plasmid DNA and incubated for 6 h at 30°C in darkness. Cells were plated on agar plates without antibiotics and kept in a climate chamber at 28°C and 50 μE m^2^s^1^. On the third day, antibiotics were added for selection pressure. After 2 weeks, single colonies appeared that were streaked on new BG11 agar plates with antibiotics for segregation six to eight times. Resulting transformants were either checked by PCR or Southern hybridization (Supplementary Figures 3–7).

### Growth Conditions

Strains were either cultivated in BG11 medium which contains 17.6 mM nitrate or alternatively in BG110 medium without nitrate that was supplemented with 5 mM arginine. Notably, 10 mM glucose was added as indicated. DCMU was added at a concentration of 10 mM. For precultures, 50 ml of BG11 medium were inoculated with cells and antibiotics in the case of mutants in 100 ml Erlenmeyer flasks on a rotary at 28°C, 50 μE/m^2^/s, and 100 rpm. After several days of growth, cultures were pelleted and washed twice in the medium of choice without antibiotics for growth experiments. Cells were inoculated into 200 ml BG-11 at an OD750 of 0.05 and placed into glass tubes with a diameter of 3.5 cm bubbled with air at 50 μE/m^2^/s at 28°C, and growth was monitored every 24 h by measuring the optical density at 750 nm as described earlier (Makowka et al., 2020). The optical density (OD) of the culture was determined by photometrical analysis (UV 2501 PC Photometer, Shimadzu, Kyoto, Japan) at 750 nm, and its data were recorded and analyzed using the affiliated software UVProbe 2.33 (Shimadzu, Kyoto, Japan). Samples were diluted with BG11 medium by 1:10 when samples showed an OD_750_ value above 0.5.

### Oxygen and Hydrogen Measurements

To measure the concentration of dissolved oxygen and hydrogen in the cultures, oxygen and hydrogen sensors from Unisense (Unisense, Aarhus, Denmark) were used according to the manufacturer's instructions. After a two-point calibration of the sensors, they were placed in the respective culture in glass tubes, and the measurement was started. Photosynthetic and respiratory activities were monitored *via* the oxygen evolution rate in the light and oxygen uptake rate in darkness. For this, calibrated sensors were put in a 25 ml culture (one sensor per culture, 3 repeats), which was illuminated for 15 min at 100 μmol photons m^−2^ s^−1^ followed by another 15 min in the darkness. For monitoring of oxygen and hydrogen concentrations during growth experiments, one hydrogen and one oxygen sensor were placed into the glass tubes with a diameter of 3.5 cm (Makowka et al., 2020). For the experiments shown in Supplementary Figure 1, one oxygen and one hydrogen sensor were placed into the glass tubes with cultures. The cultures with the sensors were incubated in dark and anoxic conditions from 0 to 0.73 h in order to prove their ability to produce fermentative hydrogen. As soon as the cells were illuminated, fermentative hydrogen was consumed. The cultures were purged with ambient air until they were saturated with O_2._ The aeration was turned off, and the cultures were left under continuous light.

### Hydrogenase Activity Measurements *via* Methyl Viologen

Hydrogenase activity was determined *via* methyl viologen (MV) as described before (Appel et al., 2020).

### Expression of HoxH mRNA

After purification, 1 μg of RNA was subjected to a DNase digest. To check if the digest was complete, a test PCR with primers specific for *rnpB* was performed. If no PCR product was found, the RNA was reverse transcribed using the High Capacity RNA-to-cDNA Kit with the MuLV reverse transcriptase (Applied Biosystems, Warrington, UK). Subsequently, equal amounts of cDNA were used for the real-time PCR with the Power SYBR^®^ Green PCR Master Mix (Applied Biosystems, Warrington, UK). In this case, the temperature program was step 1 95°C 10 min, step 2 95°C 15 s, step 3 60 °C 60 s, and additional 39 cycles between steps 2 and 3 in the PCR Cycler Rotor-Gene Q (Qiagen, Hilden, Germany). For the quantification, the 2^−Δ*ΔCT*^ method was used to analyze relative changes in transcript abundance (Livak and Schmittgen, 2001). The *C*_T_ value was determined for each sample. DNA was diluted 1:30, 1:300, and 1:3,000. The threshold was set for all samples to the normalized fluorescence 10^−1^. A typical serial dilution is shown in Supplementary Figure 8. Data were analyzed using Rotor-Gene Q Software (version 2.0.2).

The *C*_T_ value was determined and normalized to the reference gene 16S rRNA under two different conditions (in BG11 and BG11_0_ with arginine and glucose). From the *C*_T_ values, the following ratio was determined:


$$\begin{array}{l} ratio=\;\frac{2^{C_{T}\left(gene\;of\;intest\;condition\;1\right)-\;C_{T}\left(gene\;of\;interest\;condition\;2\right)}}{2^{C_{T}\left(refernence\;gene\;condition\;1\right)-\;C_{T}\left(reference\;gene\;condition\;2\right)}} \end{array}$$

and is given in the different figures.

### Protein Preparation, Protein Analysis, and Immunoblotting

Soluble protein extracts of various *Synechocystis* sp. PCC 6803 strains were generated by glass bead breakage and differential centrifugation as described (Appel et al., 2020). Protein concentrations were determined by Bradford assay (Carl Roth). Soluble protein samples were either separated on 10% (w/v) denaturing 1-D SDS-PAGE Bis/Tris gels using an MES running buffer or on 0.75 mm thick 12% (w/v) native 1-D BN-PAGE gels (Boehm et al., 2009). Prior to 1-D BN-PAGE, ß-dodecyl-D-maltoside (ß-DM) was added to a final concentration of 0.5% (w/v) from a 10% (w/v) stock to solubilize any remaining thylakoid membrane fragments and as a last step, 5% (v/v) Coomassie loading solution [750 mM ε-aminocaproic acid, 5% (w/v) Coomassie-G] was added. 2-D BN/SDS-PAGE and immunoblotting were performed as described by Appel et al. (2020). Primary antibodies used in this study were kindly provided by Prof. Peter Nixon (Imperial College, UK; purified, polyclonal antisera from rabbit against HoxE, HoxF, HoxU, HoxY, and HoxH).

### Determination of Total Protein Amount Based on the OD_750_

To determine if *Synechocystis* cells can be disrupted with similar efficiency, the protein concentration was measured in relation to the OD_750_. Therefore, liquid cultures were cultivated in glass tubes for 4 days. Then, the OD_750_ of the cultures was measured, and the culture volume that corresponded to an OD_750_ of 0.1 and 0.3 was calculated, respectively. After centrifugation for 5 min at room temperature (RT), the pellet was resuspended in 500 μl ACA buffer (750 mM ε-aminocaproic acid, 50 mM Tris-HCl pH 7.5, and 0.5 mM EDTA), and the suspension was transferred to a new 2 ml reaction tube. In the next step, precisely 0.25 g of glass beads (0.17–0.18 mm, *Sigma Aldrich, Germany*) was added to the cell suspension. The mixture was vortexed at 4°C for 2 min using the following program: 12 cycles: 10 s “ON” followed by 10 s “OFF.” The speed was set to maximum. Later, the glass beads were pelleted at 5,000 × *g* and 4°C for ~1 min. To pellet the membrane fraction, the supernatant was transferred to a new 1.5 ml reaction tube and was centrifuged again at 12,000 × *g* and 4°C for 20 min. Then, the protein concentration was determined using the Bradford assay. To calculate the number of cells that were not broken up during the method described, the samples were examined under a light microscope. To simplify the counting, a *Neubauer* counting chamber was used. These chambers are divided into nine large squares, each with a 1 mm^2^ area. The large squares are divided into 25 medium squares, and these are again divided into 16 small squares. Each of the smallest squares has an area of 0.0025 mm^2^. Four of the medium squares were counted, and the total number of intact cells was extrapolated. The counting was repeated in triplicates, and the mean value was calculated. We found that it was possible to break cells quantitatively at low OD_750_ between 0.1 and 0.3. Therefore, total protein contents were determined at these cell densities.

### Dual-KLAS/NIR

To measure the electron transfer around PSI (from plastocyanin to P700 to ferredoxin), cell suspensions were adjusted to an OD750 of 5.7 and illuminated for 600 ms with 1,350 μE/m^2^/s. This illumination was repeated 20 times with an intermittent dark period of 30 s, and the average of the 20 measurements was calculated. Deconvolution of PC, P700^+^, and ferredoxin from the original traces of the Dual-KLAS/NIR was performed as described (Theune et al., 2021). The Dual-KLAS/NIR measures at six different wavelengths in the near-infrared. From these wavelengths, four different signals are calculated. Since the absorption of the three components (i.e., plastocyanin, P700^+^, and ferredoxin) contributes to a different extent to the four signals, the proportions of oxidized plastocyanin, P700^+^, and reduced ferredoxin can be calculated by deconvolution (Klughammer and Schreiber, 2016).

### Photochemical Quenching

Photochemical quenching (qP) measurements were conducted using a Multi-Color-PAM (Multiple Excitation Wavelength, Chlorophyll Fluorescence Analyzer, Heinz Walz, Effeltrich, DE). All cultures were brought to a chlorophyll concentration of 2.5 μg/ml and independently measured at least three times each day. A volume of 2 ml of each culture were measured in a cuvette. The actinic light was adjusted to 58 μE/m^2^/s, and there were five repetitions of a sequence of 120 s of actinic light, a saturation flash (SAT), and then a dark phase of 30 s in one measurement. qP was calculated from *F*_V_/(*F*_M_-*F*_0_) (van Kooten and Snel, 1990).

### Chlorophyll Content

To measure the chlorophyll *a* content, 3 × 100 μl of culture was centrifuged at 14,000 rpm (*Centrifuge 5424, Eppendorf*) for 5 min at RT. The cell pellet was resuspended in 1 ml of 100% methanol, vortexed for 3 min, and centrifuged again at 14,000 rpm (*Centrifuge 5424, Eppendorf*) for 5 min at RT to spin down cell debris. Using a spectrophotometer, the wavelengths 665, 666, and 750 nm were measured since chlorophyll *a* absorbs at the wavelengths 440–450 nm and 650–700 nm.

According to the following formula (Lichtenthaler, 1987), the chlorophyll *a* content was calculated as follows:


$$\begin{array}{l} \text{ Chl }a\text{ content }(\text{μg/ml})=\\ \frac{\left(\text{maximum of OD}665\text{ to OD}666\right)-\text{OD}750}{0.0809}▪\;\text{dilution} \end{array}$$

### Glucose Quantification

Glucose was quantified enzymatically by measuring the evolution of NADPH photometrically as a change of absorption at 340 nm in a sample volume of 5 μl (containing <5 mM glucose) in the presence of 0.5 units hexokinase, 0.5 units glucose-6-phosphate dehydrogenase, 2 mM ATP, 2 mM NADP^+^, and 3 mM MgCl_2_ in 91.3 mM Tris-HCl buffer in a total volume of 50 μl. Absorption measurements were performed using a 96-well plate reader (Plate Reader Infinite M200Pro, Tecan, Austria).

### Arginine or Cyanophycin Extraction and Quantification

One molecule of cyanophycin contains one molecule of arginine. Therefore, the concentration of cyanophycin equals the amount of arginine. Cyanophycin quantification was modified according to the study by Elbahloul et al. (2005) (Messineo, 1966). Step 1: cyanophycin extraction: 25 ml culture (OD_750_:1.0) was collected by centrifugation (10 min, 4,000 *g*, RT), and 1 ml acetone was added to the pellet and incubated in a shaker at 1,400 *g* for 30 min. The sample was centrifuged at 13,000 *g* for 10 min, the supernatant was discarded, and the pellet was resuspended in 1.2 ml of 0.1 M HCl and incubated for 1 h at 60°C at 1,400 *g*. The sample was centrifuged at 13,000 *g* for 10 min at 4°C, the supernatant was transferred to a new reaction cup, and 300 μl of 0.1 M Tris-HCl, pH 9.0 was added. The sample was incubated at 4°C for 40 min, centrifuged at 18,000 *g* for 15 min, and the supernatant was discarded. The pellet was resuspended in 1 ml of 0.1 M HCl and used for the quantification. Arginine standards with concentrations of 0, 10, 20, 30, 40, 50, 60, and 70 μg were prepared. Step 2: cyanophycin quantification using the Sakaguchi reaction: 166 μl reagent A [300 mg KI (potassium iodide) in 100 ml distilled H_2_O] was added to 166 μl sample/standard containing between 10 and 100 μg arginine (0.057–0.5 μM, 0.1 M HCl). A volume of 500 μl of reagent B (100 ml of 5 M KOH, 2 g potassium sodium tartrate, 0.1 g 2,4-dichloro-1-naphthol, 180 ml absolute ethanol, and 0.2 ml NaClO) was added, and the reaction was incubated for 1 h at RT. A volume of 166 μl of reagent C [5%(v/v) NaClO with distilled H_2_O] was added, and the sample was incubated for exactly 10 min. The absorption at 520 nm was read immediately against a blank without arginine/cyanophycin.

### Transmission Electron Microscopy

A volume of 30 ml of each *Synechocystis* culture were harvested at 8,000 *g* for 3 min and resuspended in 2 ml of the corresponding growth medium. The concentrated cells were fixed by adding 2 ml of a solution containing 5% glutaraldehyde (GA) and 2% paraformaldehyde (FA) in 0.2 M cacodylate buffer (pH 7.3). Samples were either stored at 4°C or directly observed with a transmission electron microscope (TEM, Tecnai G2 Spirit BioTWIN, Fei Company, Thermo Fisher Scientific, MS, USA).

### Photosynthesis and Respiration Rate Measurements

The respiration and photosynthesis rates of *Synechocystis* cultures were determined as the oxygen uptake rate in darkness and oxygen evolution rate in the light, which was determined using a Unisense oxygen microsensor (Unisense, Denmark) according to the manufacturer's instruction.

## Results

### Growth Behavior of Synechocystis WT and the *ΔhoxH* Mutant Strain Under Photomixotrophic Conditions on Arginine in the Presence of Oxygen

**Figure 1:**
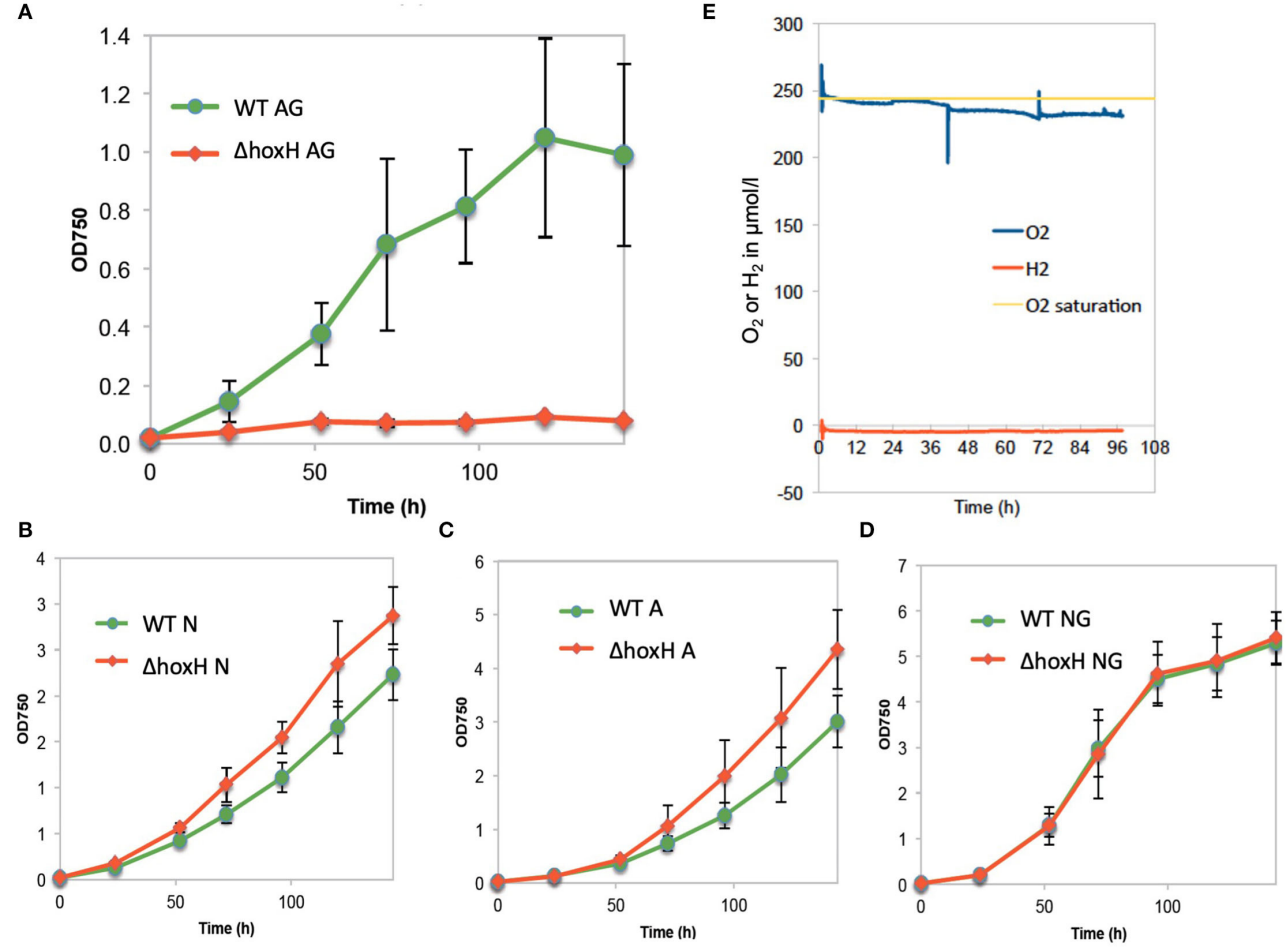
Growth of wild type (WT) and ΔhoxH under different conditions and oxygen and hydrogen concentrations in cultures on arginine and glucose in continuous light (60 μE/m2/s). **(A)** Growth in arginine and glucose (AG), **(B)** growth in nitrate (N), **(C)** growth in arginine (A), **(D)** growth in nitrate and glucose (NG), **(E)** O_2_ and H_2_ concentration in the cultures cultivated on arginine and glucose.

When WT and Δ*hoxH* were cultivated on arginine and glucose under continuous light, Δ*hoxH* displayed no growth, whereas the growth rate was similar to WT when cultured on nitrate, on arginine, or on glucose plus nitrate (Figures 1A–D).

Oxygen and hydrogen concentrations and OD_750_ were monitored in WT cells cultured on arginine and glucose for 96 h. Oxygen concentrations were close to saturation throughout the experiment, whereas, no hydrogen was detected (Figure 1E). As the cultures were purged with ambient air, in this setting, hydrogen might have been gassed out from the cultures. Therefore, hydrogen and oxygen concentrations in WT cultures on arginine and glucose were monitored during growth under continuous light without purging (Supplementary Figure 1). Again, no hydrogen was detected, whereas oxygen accumulated. These results show that oxygen is present, but no hydrogen is detected under conditions where HoxH is required for growth.

### Morphology and Metabolic State of WT and *ΔhoxH* on Arginine and Glucose

**Figure 2:**
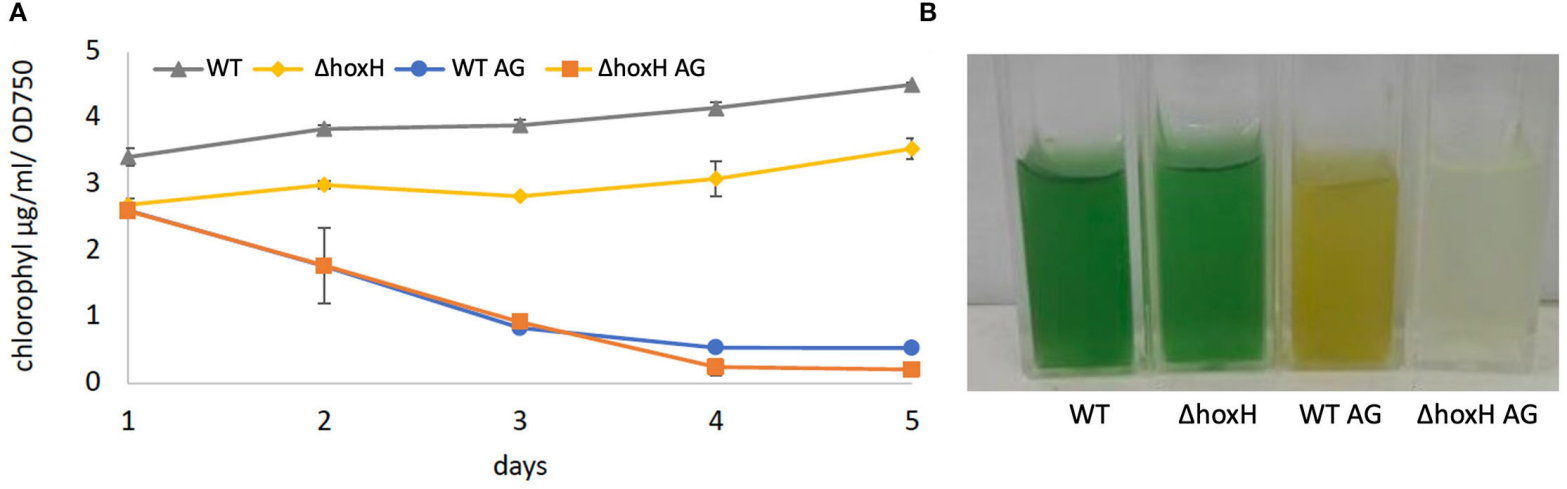
**(A)** Chlorophyll content of WT and Δ*hoxH* during growth experiments under photoautotrophic conditions and on arginine and glucose. **(B)** The appearance of strains on day 3 after inoculation.

Wild-type and Δ*hoxH* reduced their chlorophyll content relative to OD_750_ on arginine and glucose. WT cultures appeared yellow, and Δ*hoxH* cultures appeared colorless due to their low optical densities (Figure 2).

**Figure 3:**
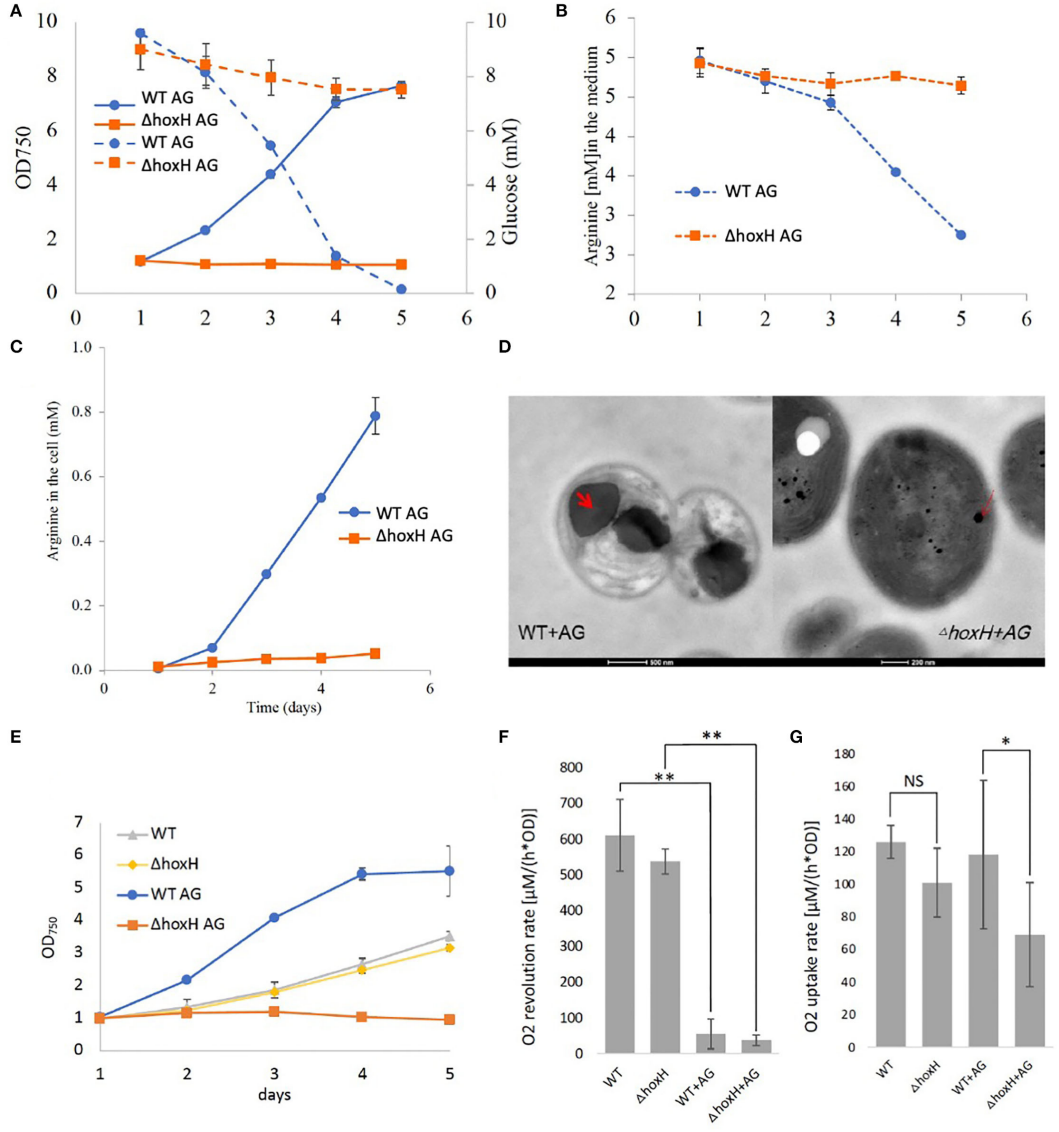
Metabolism and morphology of WT and Δ*hoxH* on arginine and glucose. **(A)** Growth (solid line) and glucose consumption (dotted line). **(B)** Arginine consumption, **(C)** Cyanophycin content of cells displayed as arginine in the cells, **(D)** Transmission electron microscopy (TEM) images of WT and Δ*hoxH* that were cultivated on arginine and glucose. Cyanophycin granules are marked with a red arrow in the WT. **(E)** Growth experiment in which **(F)** dark respiration and **(G)** photosynthesis were determined by O_2_ uptake and evolution on days 2–4. Stars on the column diagram indicate differences between mutant and WT by Tukey's HSD test (*P < 0.05, **P < 0.01). Throughout the figure ‘NS' indicates ‘not significant'. All error bars indicate standard deviation (s.d.) of four daily independent experiments.

Quantification of glucose and arginine in the medium of WT and Δ*hoxH* cultures revealed that WT cells consumed both compounds, whereas consumption by Δ*hoxH* was negligible (Figures 3A,B). In agreement with this, the WT produced and stored large amounts of cyanophycin, which is composed of arginine and aspartic acid, whereas Δ*hoxH* did not (Figure 3C). Microscopic images confirmed that cyanophycin granules accumulated in WT cells only. Furthermore, they revealed that WT cells underwent dramatic morphological changes as they degraded their thylakoid membranes in contrast to Δ*hoxH* (Figure 3D). The morphological changes observed in WT cells are well in line with earlier observations that were made on WT cells cultivated on arginine (Stephan et al., 2000). In contrast, Δ*hoxH* cells cultured on arginine and glucose looked similar to Δ*hoxH* cells cultured on nitrate. The photosynthetic activities of WT and Δ*hoxH* cells were similar on nitrate and were strongly reduced in both when cultivated on arginine and glucose (Figures 3E,F). Dark respiration in Δ*hoxH* was decreased compared with the WT, which is in line with its lower glucose and arginine consumption rates under the growth in continuous light (Figure 3G). It is remarkable that Δ*hoxH* reduces its photosynthesis and respiratory activity even though the cells look normal and healthy with thylakoid membranes and only small amounts of cyanophycin granules. In view of the fact that Δ*hoxH* cells neither consumed glucose nor arginine in large quantities, were unable to grow and showed no morphological changes, it is assumed that Δ*hoxH* cells entered a dormant-like state and were unable to adapt in the same manner as WT cells.

### Importance of Diaphorase (HoxEFU) and Hydrogenase (HoxYH) Moiety on Arginine and Glucose

As no hydrogen was detected and hydrogenase is known to be oxygen-sensitive, one explanation for the function of Hox may be that the diaphorase is critical to growth under oxic conditions on arginine and glucose. To test this, levels of HoxEFU in Δ*hoxH* cell extracts were monitored by immunoblot. We also constructed a mutant strain Δ*hoxW* that is defective in the maturase HoxW that activates HoxH (Eckert et al., 2012). This mutant should in principle contain a fully assembled and functional HoxEFU diaphorase but lack a functional HoxH.

**Figure 4:**
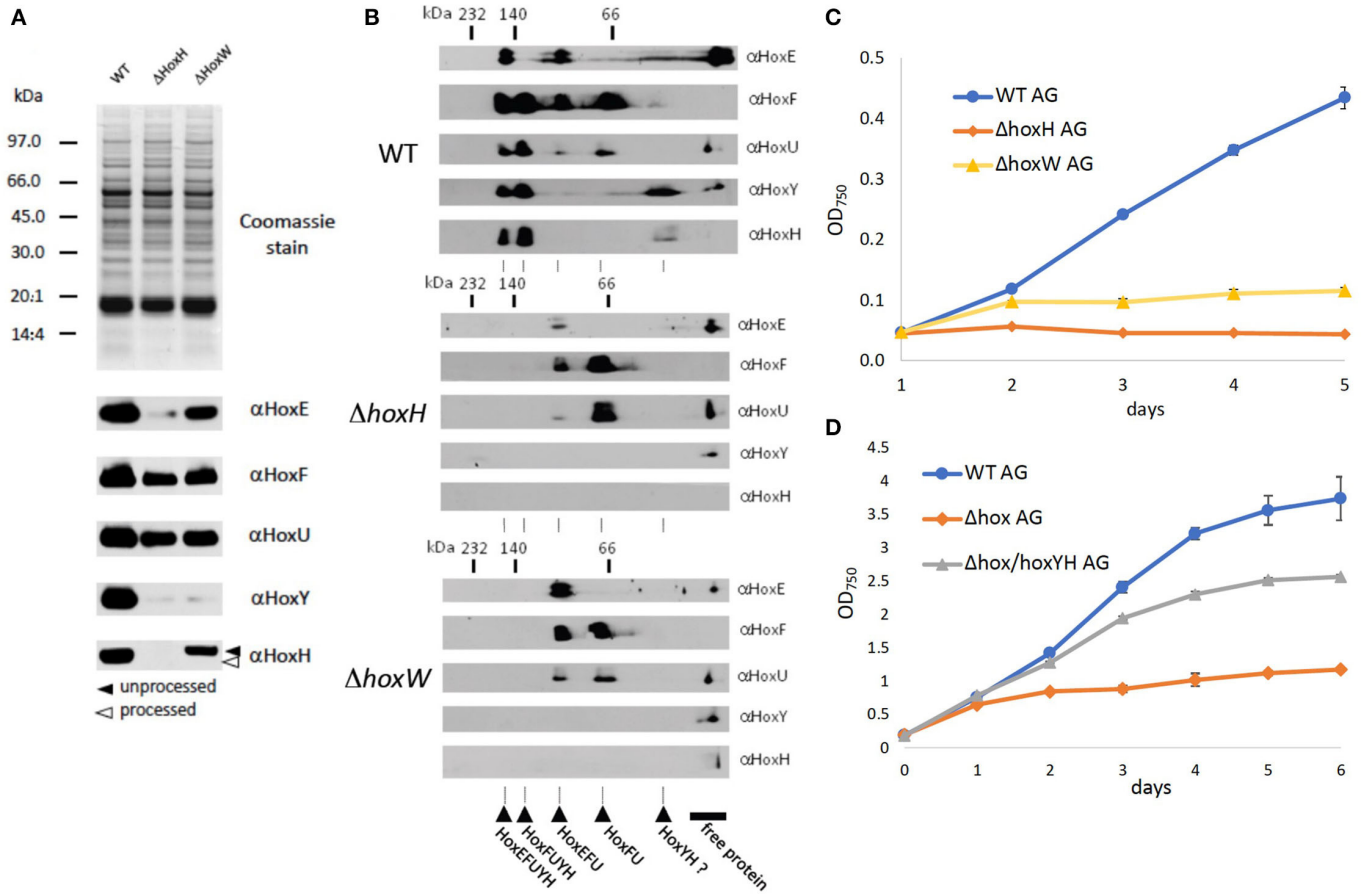
Significance of the diaphorase and the hydrogenase on arginine and glucose. **(A)** SDS page and immunoblots of WT, Δ*hoxH*, and Δ*hoxW* with antibodies against all hox subunits **(B)** 2D Blue Native PAGE of WT, Δ*hoxH*, and Δ*hoxW* with antibodies against all hox subunits. The second dimension allows to check for the assembly of complexes. **(C)** Growth of WT, Δ*hoxH*, and Δ*hoxW* on arginine and glucose. **(D)** Complementation of Δ*hox* with *hoxYH* (Δ*hox*/hoxYH). Growth of WT, Δ*hox/hoxYH*, and Δ*hox* on arginine and glucose.

Immunoblots and 2D Blue Native PAGE revealed the presence and assembly of the diaphorase HoxEFU in both the Δ*hoxH* and Δ*hoxW* cells (Figures 4A,B). HoxE levels were slightly reduced in the Δ*hoxH* background but were fully present in Δ*hoxW* cells. HoxY levels were very low in both mutants, whereas HoxH was absent in Δ*hoxH* and only present in its unprocessed form in Δ*hoxW* cells (Figure 4A). In the WT the most prominent complexes that are visible in the second dimension are HoxEFUYH and HoxFUYH (Figure 4B). These complexes are missing in *hoxH* and *hoxW* deletion strains. However, complexes that contain the diaphorase subunits only, namely HoxEFU and HoxFU, are present in the WT and in both mutants.

As the diaphorase was apparently present and assembled in Δ*hoxW*, we tested the growth of this mutant on arginine and glucose and found it to be similar but slightly better than the growth of Δ*hoxH* cells (Figure 4C). In addition, when a Δ*hox* mutant, in which *hoxEFUYH* was deleted, was complemented with *hoxYH*, growth improved considerably, but not to WT levels (Figure 4D). These results indicate that the hydrogenase (HoxYH) is the critical component and that the diaphorase (HoxEFU) alone cannot support the growth of arginine and glucose. However, we cannot rule out that the holoenzyme of HoxEFUYH is required for the proper functioning of the diaphorase *in vivo*. The fact that the diminished growth of Δ*hox* (Δ*hoxEFUYH*) could be partly rescued by the introduction of *hoxYH*, furthermore, indicates that the hydrogenase indeed fulfills an important function without the diaphorase under these conditions but that, in contrast, the holoenzyme HoxEFUYH is required for WT-like growth on arginine and glucose.

### Transcription and Expression of the Hydrogenase Under Mixotrophic Conditions on Arginine

As the growth of the WT and Δ*hoxH* strains deviates from each other within the first day of cultivation, mRNA was isolated from cells cultured on nitrate (BG11) as control and on arginine and glucose (BG11_0_AG) at 12, 24, 36, 48, and 72 h after inoculation. In cells cultured on arginine and glucose, the transcription of *hoxH* was upregulated 2-fold after 24 h and 5-fold after 36 h from inoculation (Supplementary Figure 2A). The expression levels of the *hoxEFUYH* operon 24 h after inoculation on arginine and glucose revealed that the entire *hox* operon was upregulated (Supplementary Figure 2B). Despite increased transcription, protein levels of Hox subunits (HoxE, F, U, Y, H) appeared to be unaffected (Supplementary Figures 2C,D).

### Does the Hydrogenase Work as an Electron Valve for Photosynthesis on Arginine and Glucose?

**Figure 5:**
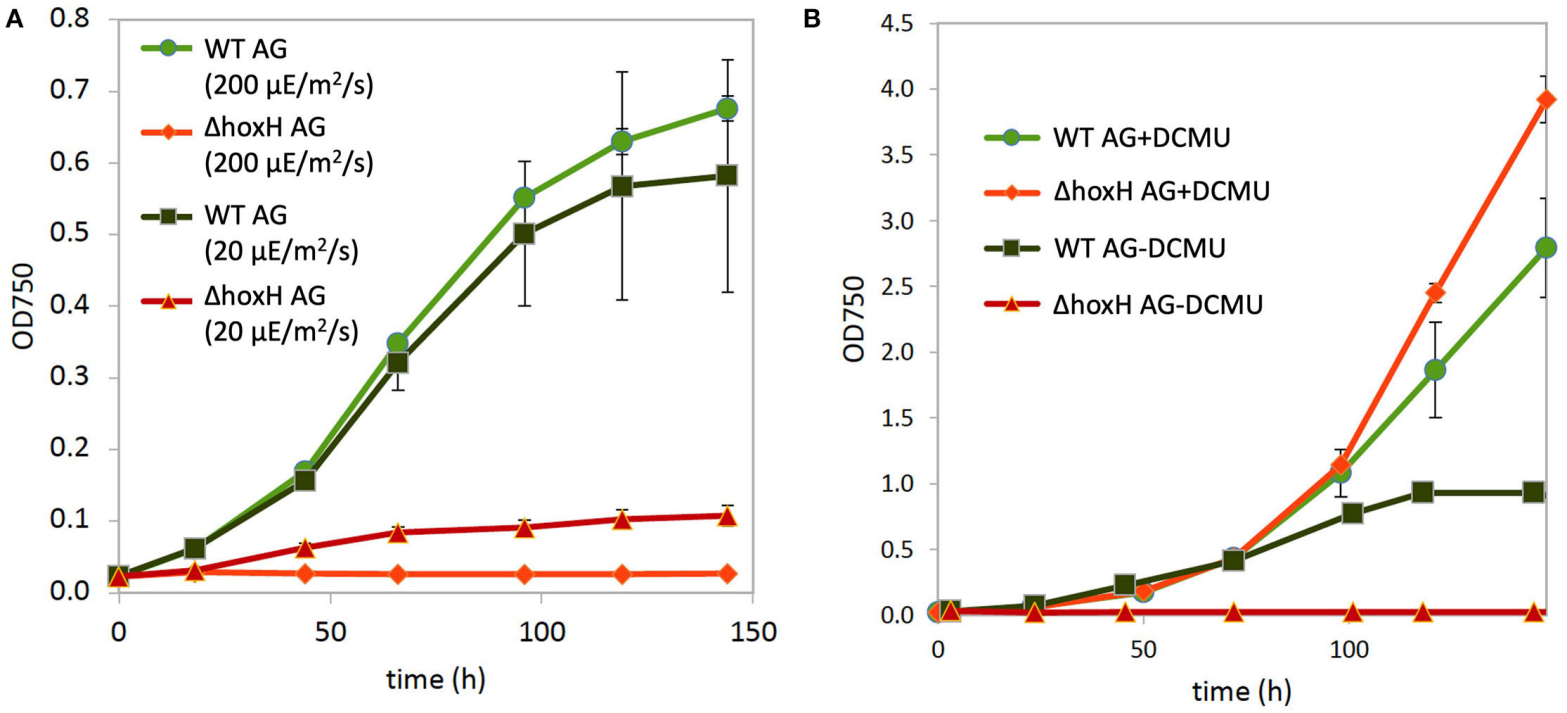
Growth of WT and Δ*hoxH* on arginine and glucose. **(A)** Growth at high light (200 μE/m^2^/s) and low light (20 μE/m^2^/s). **(B)** Growth at medium light (60 μE/m^2^/s) in the absence and presence of the inhibitor DCMU, which blocks electron transfer from PSII to the plastoquinone (PQ) pool.

Three main sources feed electrons into the PQ pool of the photosynthetic electron transport chain in cultures that are cultivated on arginine and glucose as follows: water splitting at PSII, the oxidation of the carbon skeleton of arginine, and the oxidation of glucose. This might lead to an over-reduced photosynthetic electron transfer chain with a highly reduced PQ pool. Therefore, the hydrogenase might work as an electron valve under these conditions. To test this, WT and Δ*hoxH* cells were cultivated on arginine and glucose under high light (200 μE/m^2^/s) and under low light (20 μE/m^2^/s) in order to determine if low light relieves the reductive stress to improve the growth of Δ*hoxH*. The WT strain grew well under both light intensities, whereas Δ*hoxH* which did not grow at all at 200 μE/m^2^/s but was able to grow under low light at 20 μE/m^2^/s (Figure 5A).

**Figure 6:**
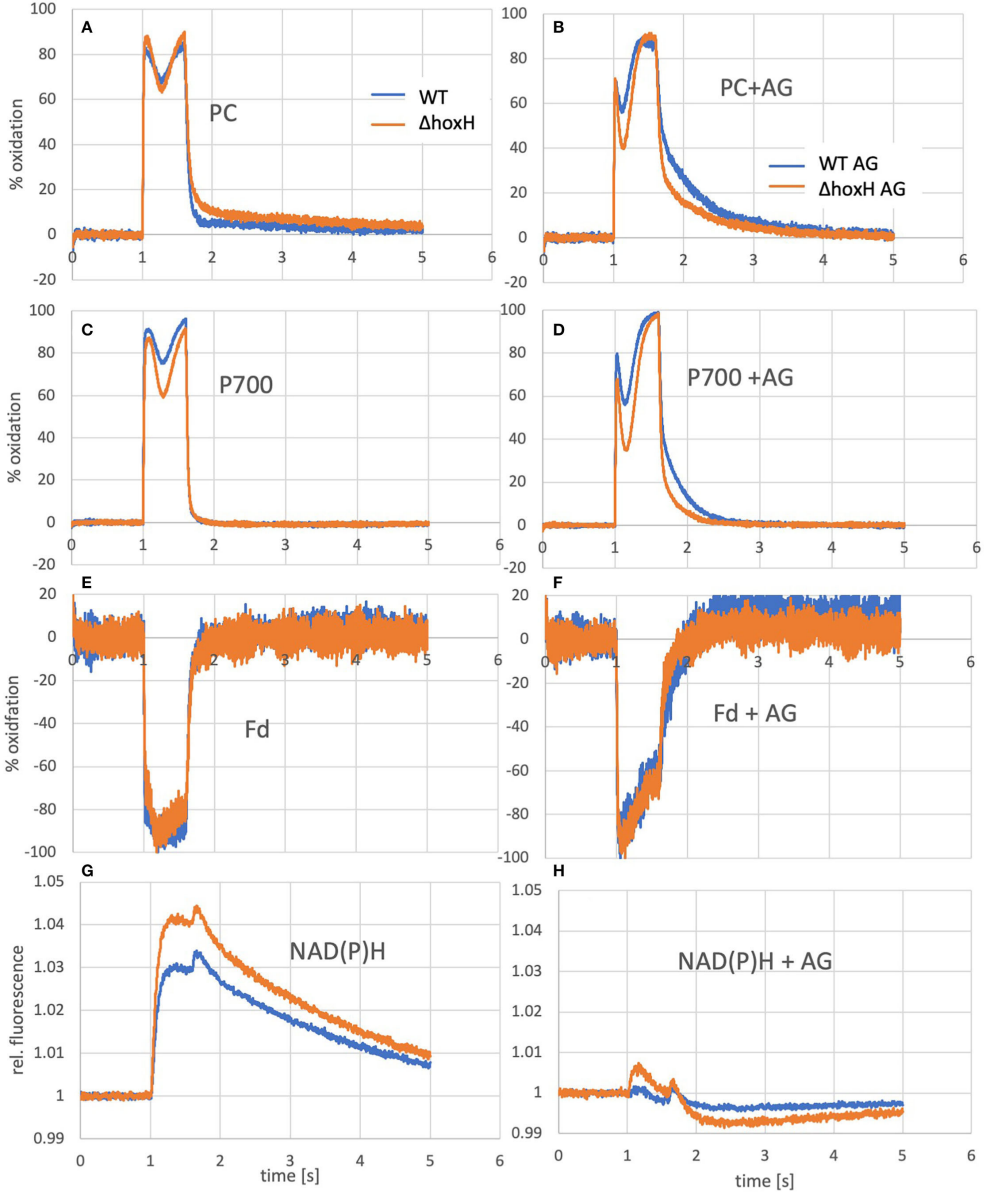
Oxidation of PC and P700 and reduction of ferredoxin and NADPH of WT and Δ*hoxH* cultures as measured by the Dual-KLAS/NIR. On the left **(A, C, E, G)**, curves of cultures grown photoautotrophically are shown and on the right **(B, D, F, H)**, the same cultures are shown that were measured a few minutes after the addition of arginine and glucose.

The addition of DCMU, which blocks electron transfer from PSII to the PQ pool, enhanced the growth of WT under medium light intensities (60 μE/m^2^/s), and the growth of Δ*hoxH* was completely rescued to WT levels (Figure 6B). These data show that the requirement for the hydrogenase on arginine and glucose is related to electron transport during photosynthesis.

**Figure 7:**
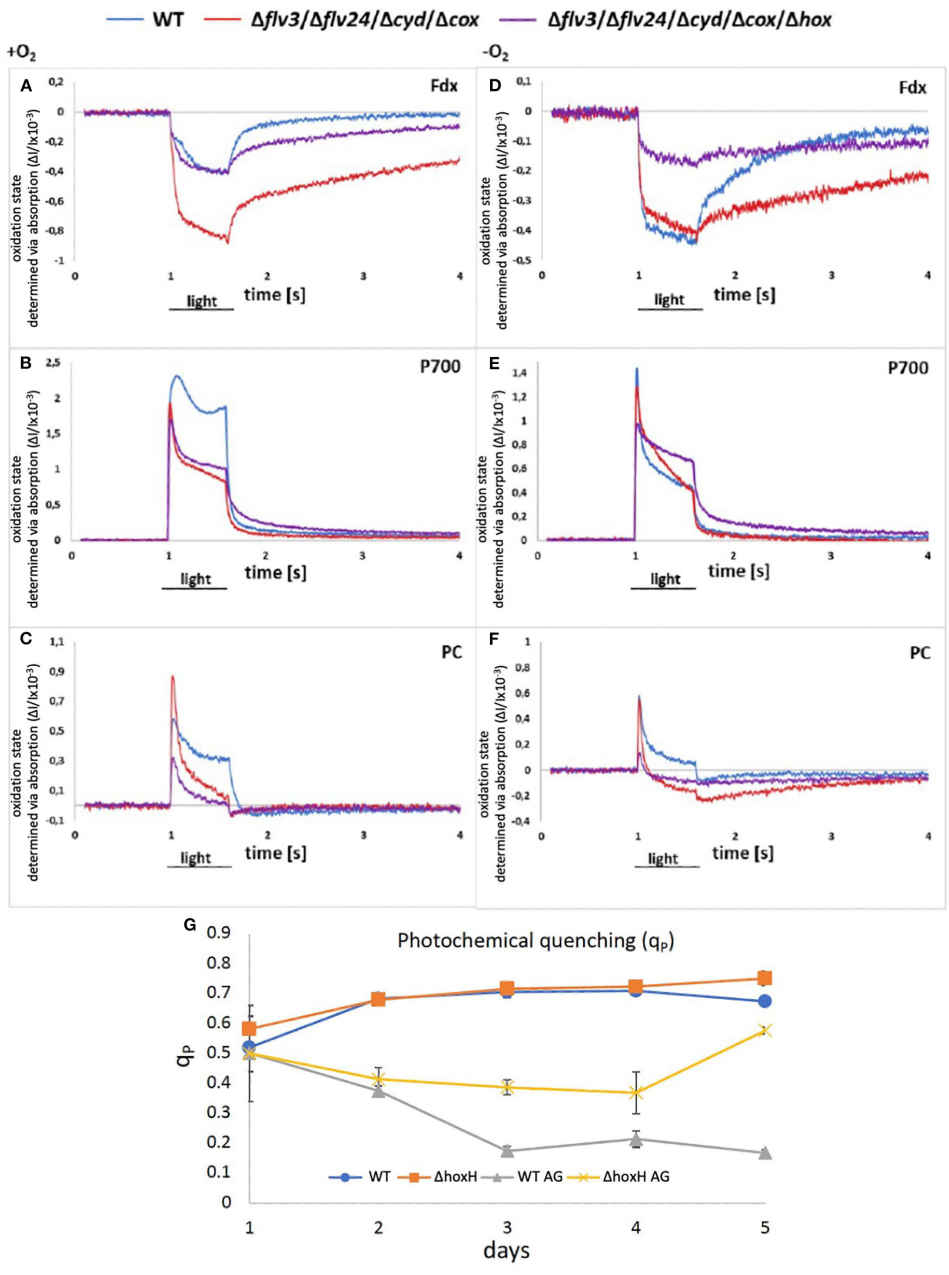
**(A–F)** Redox states of components of the photosynthetic electron chain on arginine and glucose in the presence and absence of oxygen in WT, Δ*flv24*Δ*flv3*Δ*cox*Δ*cyd, and* Δ*flv24*Δ*flv3*Δ*cox*Δ*cyd*Δ*hox*. The oxidation state was measured as absorption change (ΔI/I ×10^−3^). Positive values show oxidation and negative values show a reduction of components. **(G)** Photochemical quenching (q_P_) in WT and Δ*hoxH* on nitrate (WT and Δ*hoxH*) and on arginine and glucose (WT AG and Δ*hoxH* AG).

To test if the hydrogenase has a direct and immediate impact on the photosynthetic electron transfer chain, the reduction and oxidation of plastocyanin (PC), P700, and ferredoxin were monitored upon the addition of arginine and glucose in WT and Δ*hoxH via* Dual-KLAS/NIR (Figure 6). PC and P700 are oxidized in WT and Δ*hoxH* at the onset of illumination (Figures 6A–D). Under photoautotrophic conditions, the first oxidation peak is reached ~100 ms into the light pulse and is followed by a transient phase of stronger reduction of PC and P700 (Figure 6A). The reduction is caused by electrons coming in from PSII due to the reduced electron acceptor pool (Fdx and NADP^+^) of PSI. After another 200 ms, this reduction phase reverts, and oxidation predominates again before the light is turned off after 600 ms (Theune et al., 2021). This second oxidation phase is governed by the activity of the flavodiiron proteins (mainly Flv1/3) that catalyze oxygen reduction at the acceptor side of PSI. In the absence of the flavodiiron proteins, this phase is missing (refer to Figure 7B) (SÈtif et al., 2020). In Δ*hoxH*, the reduction phase is faster and deeper for PC and P700 in contrast to the WT. This effect is more pronounced in the presence of arginine and glucose (Figures 6B,D). The first peak of oxidation becomes narrower and is reached after only 35 ms before reduction becomes stronger. Δ*hoxH* shows a deeper reduction trough in the light phase and a faster reduction in the dark phase. The mutant obviously suffers from a stronger acceptor side limitation. This is noticeable when comparing the reduction curves of NAD(P)H (Figures 6G,H). Under autotrophic conditions, NAD(P)H fluorescence shows a large increase in illumination, but in the presence of arginine and glucose, this peak is negligible. This indicates that the NAD(P)H pool is already mainly reduced on arginine and glucose. The same was observed for the ferredoxin pool, which was instantly fully reduced upon illumination on arginine and glucose (Figures 6E,F). Taken together, these data show that the hydrogenase works as a transient electron valve in an immediate response to the addition of organic carbon and nitrogen in the presence of oxygen.

However, as no hydrogen was traceable, the hypothesis was put forward that the hydrogenase might release electrons from the photosynthetic electron transport chain by reducing oxygen instead of protons. According to the current understanding, Ni and Fe in the active site of the hydrogenase are inactivated in the presence of oxygen by the formation of a bridging hydroxy group which prevents H_2_-turnover (Pandelia et al., 2010; McIntosh et al., 2011; Caserta et al., 2020). The oxygen tolerance of the soluble NiFe-hydrogenase of *Ralstonia eutropha* is based on its oxidase activity which reactivates the enzyme by reducing the bound hydroxyl group to water (Lauterbach and Lenz, 2013). Based on this finding, the hypothesis was put forward that the oxygen-sensitive NiFe-hydrogenase of *Synechocystis* might also function as an oxidase and switch between H_2_ production under anaerobic conditions and oxygen reduction under aerobic conditions. To test this hypothesis, two mutants were constructed. The first one lacked all of the terminal oxidases that are associated with the thylakoid membrane, namely, the respiratory terminal oxidase cytochrome C oxidase (*cyd*) and quinol oxidase (*cox*) but retained the alternative respiratory oxidase (*arto; ctaII*). In addition, flavodiiron proteins, namely, Flv2, Flv4, and Flv3, which have the potential to reduce oxygen, were deleted (Shimakawa et al., 2014; Brown et al., 2019). This resulted in the mutant strain Δ*flv24*Δ*flv3*Δ*cox*Δ*cyd*. The second mutant was the same as the first but also lacked the *hox* operon (*hoxEFUYH*), yielding the mutant strain Δ*flv24*Δ*flv3*Δ*cox*Δ*cyd*Δ*hox* (note that in this strain, the entire *hox* operon instead of *hoxH* was deleted). The redox status of photosynthetic components [plastocyanin (PC), P700 in PSI, and ferredoxin] were measured in these mutants and compared with the WT strain cultured on arginine and glucose on the second day after inoculation in the absence and in the presence of oxygen *via* Dual-KLAS/NIR (Figure 7). If the hydrogenase is functioning as an oxidase and transferring surplus electrons to oxygen, it was expected that the deletion of *hoxEFUYH* would result in more reduced photosynthetic components in the presence of oxygen. However, the ferredoxin pool and P700 were more reduced in Δ*flv24*Δ*flv3*Δ*cox*Δ*cyd* in comparison to Δ*flv24*Δ*flv3*Δ*cox*Δ*cyd*Δ*hox* indicating that acceptor side limitation at PSI was more severe in the presence of the hydrogenase (Figures 7A–D). Thus, our measurements show that the hydrogenase does not function as an oxidase.

We then examined the PSII fluorescence properties of WT and Δ*hoxH* cells cultured over a 5-day period on nitrate, or on arginine plus glucose, in order to monitor the redox state of the PQ pool. Both strains exhibited a similar PQ pool redox poise when cultivated on nitrate. When cultured on arginine plus glucose, the PQ pools of both strains were more reduced than in cells cultured on nitrate, becoming more pronounced in WT cells later in growth (Figure 7G). These data are well in line with the Dual-KLAS/NIR measurements during the growth on organic carbon and nitrogen in WT, Δ*flv24*Δ*flv3*Δ*cox*Δ*cyd*, and Δ*flv24*Δ*flv3*Δ*cox*Δ*cyd*Δ*hox* (Figures 7A–F). The fact that components of the photosynthetic electron transfer chain were more reduced in the WT in comparison to Δ*hoxH* on arginine and glucose (Figure 7G) is comprehensible considering that Δ*hoxH* did neither metabolize arginine nor glucose in large amounts and furthermore paused growth (Figure 3). Taken together, the results indicate that in the immediate response to the growth on arginine and glucose, the hydrogenase functions to dissipate electrons (Figure 6), however, in the long run for hours and days, hydrogenase supports feeding electrons into the PQ pool under these conditions, suggesting it has a dual function.

### Significance of Components of the Respiratory Electron Chain for Growth on Arginine and Glucose

**Figure 8:**
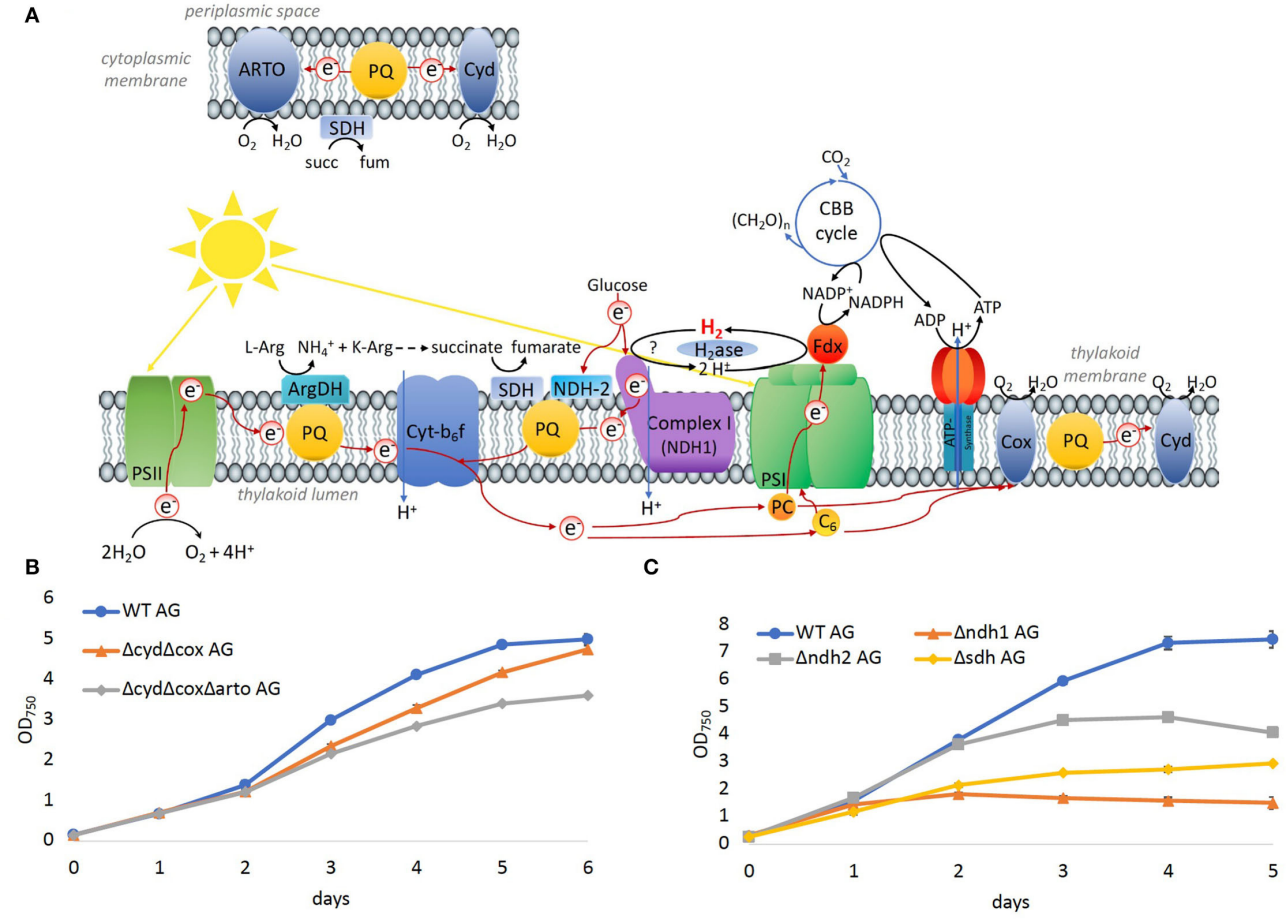
Significance of the respiratory electron chain for growth on arginine and glucose. **(A)** Overview of photosynthetic and respiratory electrons chains in the thylakoid and cytoplasmic membrane. **(B)** Growth of WT and mutants in which terminal oxidases were deleted on arginine and glucose. **(C)** Growth of WT and dehydrogenases, that feed electrons into the PQ pool on arginine and glucose.

To evaluate the significance of components of the respiratory electron transfer chain for the growth on arginine and glucose, additional deletion mutants were constructed and tested. *Synechocystis* possesses three respiratory terminal oxidases: the quinol oxidase (*cox*) is localized in thylakoid membranes, cytochrome C oxidase (*cyd*) is localized in the thylakoid and cytoplasmic membranes, and the alternative respiratory oxidase (*arto*) is restricted to the cytoplasmic membranes (Figure 8A). Deletion of both thylakoid-associated oxidases (Δ*cyd*Δ*cox*) or all three of the terminal oxidases (Δ*cyd*Δ*cox*Δ*arto*) resulted in mutants that were still able to grow on arginine and glucose albeit at impaired rates (Figure 8B).

Three main respiratory dehydrogenases feed electrons from glucose oxidation into the PQ pool in *Synechocystis*: photosynthetic complex I (NDH-1) accepts electrons from reduced ferredoxin (Schuller et al., 2019), NDH-2 accepts electrons from NADH, and the succinate dehydrogenase (SDH) catalyzes the oxidation from succinate to fumarate. In the Δ*ndh2* mutant strain, all three homologs *ndbA/ndbB/ndbC* of the NADH dehydrogenase-like complex 2 (NDH2; *slr0851, slr1743*, and *sll1484*) were deleted. In the Δ*sdh* mutant, the two subunits *sdh1* and *sdh2* of the succinate dehydrogenase (SDH; *sll1625*, and *sll0823*)) were deleted. Additionally, in the Δ*ndh1* mutant, the subunits *ndh-D1* and *ndh-D2* of complex 1 (NDH1; *slr0331*, and *slr1291*) were deleted. Growth of all three mutant strains was impaired on arginine and glucose (Figure 8C), where the severest impairment was observed for Δ*ndh1* followed by Δ*sdh*. Thus, the terminal oxidases appeared to be dispensable for growth on arginine and glucose, whereas SDH has a significant role, and hydrogenase and NDH-1 are essential.

## Discussion

An obvious question that suggests itself is why the bidirectional NiFe-hydrogenase, which is an oxygen-sensitive enzyme, is widespread in organisms that produce oxygen and rarely encounter anoxic conditions. Its presence has been regarded as a leftover from ancient times. However, the ancestors of cyanobacteria possessed several NiFe- and FeFe-hydrogenases (types 2a, 3b, 3c, 3d, and 4), whereas cyanobacteria kept only two NiFe-hydrogenases: the uptake NiFe-hydrogenase (type 2a) in association with N_2_ fixation and the bidirectional NiFe-hydrogenase (type 3d) (Vignais and Billoud, 2007). The advantage for the uptake of hydrogenase, which consumes H_2_, which is a by-product of N_2_ fixation, is apparent. But why was the bidirectional enzyme kept? Cyanobacteria in microbial mats, in fact, encounter anoxic conditions at night due to intense respiration within the microbial mat community and produce fermentative hydrogen in darkness and photohydrogen on illumination (Burow et al., 2012a,b; Bolhuis et al., 2014; Nielsen et al., 2015). The function of the cyanobacterial bidirectional NiFe-hydrogenase is thus well-understood in microbial mats. However, the enzyme is also widespread in cyanobacteria from surface waters that stay oxygenated also at night (Barz et al., 2010; Beimgraben et al., 2014; Greening et al., 2015). Remarkably, it is absent from oligotrophic habitats as the open ocean but is found in freshwater and coastal waters that have a higher load of nutrients and occasionally encounter phytoplankton blooms (Barz et al., 2010). Excretion and lysis of organisms in senescent phytoplankton blooms and the bacterial breakdown of organic carbon to shorter sugars create mixotrophic conditions in these habitats that are typically accompanied by increased levels of dissolved organic nitrogen as arginine (Wetz and Wheeler, 2003, 2004; Teeling et al., 2012). The replacement of nitrate with arginine and addition of glucose in this study thus somewhat mimics the situation of a senescent phytoplankton bloom in habitats where cyanobacterial NiFe-hydrogenases are found. The presented data indicate strongly that the hydrogenase has a function under oxic conditions which are most likely not based on hydrogen turnover but linked to photosynthesis and respiration to support a metabolic switch for growth in the presence of organic carbon and nitrogen. Please note that the concentrations of glucose and arginine that were utilized in this study are above those levels found in nature. We thus created artificial laboratory conditions so that the comparison with natural conditions must be performed cautiously. We did not find any evidence for the hypothesis that the hydrogenase works as an oxidase or functions as an electron valve during long-term growth. Rather, our results indicate that the hydrogenase supports to feed electrons into the photosynthetic electron transport chain. Since the Δ*hoxH* mutant does not consume significant amounts of glucose and arginine, it is not clear whether the hydrogenase is actively involved in feeding electrons into the photosynthetic electron transport chain in the WT, or if its absence in Δ*hoxH* rather prevents a metabolic switch and thereby results in less reduced photosynthetic components. The functional significance of hydrogenase is most likely linked to the tuning of the light reactions of photosynthesis (Figure 5). For example, *Synechocystis* possesses various complexes that fine-tune the intersystem electron transport chain including flavodiiron proteins, respiratory dehydrogenases, and terminal oxidases. Our results in this study demonstrate that most of these were dispensable for the growth on arginine and glucose and raise the question as to why hydrogenase is essential.

*In-vitro* experiments have revealed that the hydrogenase HoxEFU diaphorase sub-complex is able to exchange electrons between NAD(H), NAD(P)H, several ferredoxins and flavodoxin (Artz et al., 2020). This is a unique property of HoxEFU in having reactivity with more than four redox carriers. In contrast, FNR shuttles electrons between NADPH and ferredoxin, and the transhydrogenase (PntAB) transfers electrons from NADH to NADP^+^ (Kämäräinen et al., 2016). Although the hydrogenase catalytic subunits, HoxYH, were not required for the HoxEFU activity with redox carriers *in vitro*, it cannot be ruled out that this function requires the presence of the HoxYH subunits *in vivo*. The data presented in this study add another dimension to Hox function by demonstrating that the hydrogenase subunit HoxH, but not the hydrogen reactivity of HoxH, is required for electron exchange with the PQ pool in cells grown on arginine and glucose. As the photosynthetic complex I was likewise required for growth on organic carbon and nitrogen, it is possible that the hydrogenase transfers electrons *via* NDH-1 into the PQ pool. This idea is further supported by the fact that respiration was diminished in Δ*hoxH* cells in comparison to the WT cells (Figure 3F). It has been shown that the cyanobacterial NDH-1 complex accepts electrons exclusively from reduced ferredoxin (Schuller et al., 2019). It is conceivable that the Hox hydrogenase complex might mediate an input of electrons *via* the NDH-1 complex into the photosynthetic electron transport chain and thereby tune photosynthesis and respiration in the presence of organic carbon and nitrogen. However, this idea requires further experimental support.

## Conclusion

The cyanobacterial bidirectional NiFe-hydrogenase of *Synechocystis* is required for growth on organic carbon and nitrogen in the presence of oxygen and fulfills a function apart from hydrogen turnover. Its presence has an impact on the redox states of photosynthetic components in the presence of oxygen. It acts as an electron valve as an immediate response to the supply of arginine and glucose. The enzyme does not work as an oxidase and it does not have the role to dissipate electrons during long-term growth. Rather, hydrogenase supports the input of electrons into the photosynthetic electron transport chain. In the presence of the hydrogenase, *Synechocystis* performs a metabolic switch, which allows cells to grow on arginine and glucose but enters a dormant-like state in its absence. The exact manner by which hydrogenase influences the thylakoid membrane's electron transport chain remains unresolved. Our observations support a model for the more global occurrence of a bidirectional NiFe-hydrogenase in cyanobacteria that were found predominantly in oxygenated surface waters of nutrient-rich habitats. Its unique ability to shuttle electrons between NAD(H), NADP(H), ferredoxins, and flavodoxin might help to mediate an input of electrons *via* the NDH-1 complex into the photosynthetic electron chain. However, this view requires further experimental evidence.

## Data Availability Statement

The original contributions presented in the study are included in the article/Supplementary Material, further inquiries can be directed to the corresponding author/s.

## Author Contributions

HB, YW, JC, VH, JA, MB, SL, and MT performed experiments. HB, YW, JA, and KG contributed to conception and design of the study. HB, YW, JC, VH, JA, MB, SL, MT, PK, and KG analyzed and interpreted data. HB, YW, VH, and KG wrote the first draft of the manuscript. All authors contributed to manuscript revision, read, and approved the submitted version.

## Funding

This study was supported by grants from the China Scholarship Council (CSC) (Grant # 201406320187), the German Ministry of Science and Education (BMBF FP309), and the German Science Foundation (DFG GU1522/2-1, GU1522/5-1, and FOR2816).

## Conflict of Interest

The authors declare that the research was conducted in the absence of any commercial or financial relationships that could be construed as a potential conflict of interest.

## Publisher's Note

All claims expressed in this article are solely those of the authors and do not necessarily represent those of their affiliated organizations, or those of the publisher, the editors and the reviewers. Any product that may be evaluated in this article, or claim that may be made by its manufacturer, is not guaranteed or endorsed by the publisher.

## References

[B1] Appel J. Hueren V. Boehm M. Gutekunst K. (2020). Cyanobacterial *in vivo* solar hydrogen production using a photosystem I–hydrogenase (PsaD-HoxYH) fusion complex. Nat. Energy 5, 458–467. 10.1038/s41560-020-0609-6

[B2] Appel J. Phunpruch S. Steinmüller K. Schulz R. (2000). The bidirectional hydrogenase of Synechocystis sp. PCC 6803 works as an electron valve during photosynthesis. Arch. Microbiol. 173, 333–338. 10.1007/s002030000139 10896211

[B3] Artz J. H. Tokmina-Lukaszewska M. Mulder D. W. Lubner C. E. Gutekunst K. Appel J.. (2020). The structure and reactivity of the HoxEFU complex from the cyanobacterium Synechocystis sp. PCC 6803. J. Biol. Chem. 295, 9445–9454. 10.1074/jbc.RA120.013136 32409585 PMC7363133

[B4] Barz M. Beimgraben C. Staller T. Germer F. Opitz F. Marquardt C.. (2010). Distribution analysis of hydrogenases in surface waters of marine and freshwater environments. PLoS ONE 5, e13846. 10.1371/journal.pone.0013846 21079771 PMC2974642

[B5] Beimgraben C. Gutekunst K. Opitz F. Appel J. (2014). hypD as a marker for [NiFe]-hydrogenases in microbial communities of surface waters. Appl. Environ. Microbiol. 80, 3776. 10.1128/AEM.00690-14 PMC4054138 24727276

[B6] Boehm M. Nield J. Zhang P. Aro E.-M. Komenda J. Nixon P. J. (2009). Structural and mutational analysis of band 7 proteins in the cyanobacterium Synechocystis sp. strain PCC 6803. J. Bacteriol. 191, 6425–6435. 10.1128/JB.00644-09 19684140 PMC2753028

[B7] Bolhuis H. Cretoiu M. S. Stal L. J. (2014). Molecular ecology of microbial mats. FEMS Microbiol. Ecol. 90, 335. 10.1111/1574-6941.12408 25109247

[B8] Brown K. A. Guo Z. Tokmina-Lukaszewska M. Scott L. W. Lubner C. E. Smolinski S.. (2019). The oxygen reduction reaction catalyzed by Synechocystis sp. PCC 6803 flavodiiron proteins. Sustain. Energy Fuels 3, 3191–3200. 10.1039/C9SE00523D

[B9] Burow L. C. Woebken D. Bebout B. M. McMurdie P. J. Singer S. W. Pett-Ridge J.. (2012a). Hydrogen production in photosynthetic microbial mats in the Elkhorn Slough estuary, Monterey Bay. ISME J. 6, 863–874. 10.1038/ismej.2011.142 22011721 PMC3309353

[B10] Burow L. C. Woebken D. Marshall I. P. G. Lindquist E. A. Bebout B. M. Prufert-Bebout L.. (2012b). Anoxic carbon flux in photosynthetic microbial mats as revealed by metatranscriptomics. ISME J. 7, 1–13. 10.1038/ismej.2012.150 23190731 PMC3603402

[B11] Burroughs N. J. Boehm M. Eckert C. Mastroianni G. Spence E. M. Yu J.. (2014). Solar powered biohydrogen production requires specific localization of the hydrogenase. Energy Environ. Sci. 7, 3791–3800. 10.1039/C4EE02502D 26339289 PMC4535174

[B12] Caserta G. Pelmenschikov V. Lorent C. Tadjoung Waffo A. F. Katz S. Lauterbach L.. (2020). Hydroxy-bridged resting states of a [NiFe]-hydrogenase unraveled by cryogenic vibrational spectroscopy and DFT computations. Chem. Sci. 12, 2189–2197. 10.1039/D0SC05022A 34163984 PMC8179317

[B13] Cournac L. Guedeney G. Peltier G. Vignais P. M. (2004). Sustained photoevolution of molecular hydrogen in a mutant of *Synechocystis* sp. strain PCC 6803 deficient in the Type I NADPH-dehydrogenase complex. J Bacteriol. 186, 1737–1746. 10.1128/JB.186.6.1737-1746.2003 14996805 PMC355973

[B14] Dutta I. Vermaas W. F. J. (2016). The electron transfer pathway upon H2 oxidation by the NiFe bidirectional hydrogenase of *Synechocystis* sp. PCC 6803 in the light shares components with the photosynthetic electron transfer chain in thylakoid membranes. Int. J. Hydrogen Energy 41, 11949–11959. 10.1016/j.ijhydene.2016.01.172

[B15] Eckert C. Boehm M. Carrieri D. Yu J. Dubini A. Nixon P. J.. (2012). Genetic analysis of the hox hydrogenase in the cyanobacterium *Synechocystis* sp. PCC 6803 reveals subunit roles in association, assembly, maturation, and function. J. Biol. Chem. 287, 43502–43515. 10.1074/jbc.M112.392407 23139416 PMC3527937

[B16] Elbahloul Y. Krehenbrink M. Reichelt R. Steinbüchel A. (2005). Physiological conditions conducive to high cyanophycin content in biomass of *Acinetobacter calcoaceticus* strain ADP1. Appl. Environ. Microbiol. 71, 858–866. 10.1128/AEM.71.2.858-866.2005 15691941 PMC546767

[B17] Flores E. Herrero A. (1994). “Assimilatory nitrogen metabolism and its regulation,” in The Molecular Biology of Cyanobacteria, ed D. A. Bryant (Dordrecht: Springer Netherlands), 487–517.

[B18] Forchhammer K. Selim K. A. (2020). Carbon/nitrogen homeostasis control in cyanobacteria. FEMS Microbiol. Rev. 44, 33–53. 10.1093/femsre/fuz025 31617886 PMC8042125

[B19] Gibson D. G. Young L. Chuang R.-Y. Venter J. C. Hutchison C. A. Smith H. O. (2009). Enzymatic assembly of DNA molecules up to several hundred kilobases. Nat. Methods 6, 343–345. 10.1038/nmeth.1318 19363495

[B20] Greening C. Biswas A. Carere C. R. Jackson C. J. Taylor M. C. Stott M. B.. (2015). Genomic and metagenomic surveys of hydrogenase distribution indicate H2 is a widely utilised energy source for microbial growth and survival. ISME J. 10, 1–17. 10.1038/ismej.2015.153 26405831 PMC4817680

[B21] Gutekunst K. Chen X. Schreiber K. Kaspar U. Makam S. Appel J. (2014). The Bidirectional NiFe-hydrogenase in Synechocystis sp. PCC 6803 is reduced by flavodoxin and ferredoxin and is essential under mixotrophic, nitrate-limiting conditions. J. Biol. Chem. 289, 1930–1937. 10.1074/jbc.M113.526376 24311779 PMC3900943

[B22] H. K. Lichtenthaler (1987). Chlorophylls and carotenoids: Pigments of photosynthetic biomembranes. Methods in Enzymol. 148, 350–382. 10.1016/0076-6879(87)48036-1

[B23] Kämäräinen J. Huokko T. Kreula S. Jones P. R. Aro E.-M. Kallio P. (2016). Pyridine nucleotide transhydrogenase PntAB is essential for optimal growth and photosynthetic integrity under low-light mixotrophic conditions in *Synechocystis* sp. PCC 6803. *New Phytol*. 214. 10.1111/nph.14353 27930818

[B24] Klughammer C. Schreiber U. (2016). Deconvolution of ferredoxin, plastocyanin, and P700 transmittance changes in intact leaves with a new type of kinetic LED array spectrophotometer. Photosyn. Res. 128, 195–214. 10.1007/s11120-016-0219-0 26837213 PMC4826414

[B25] Lauterbach L. Lenz O. (2013). Catalytic production of hydrogen peroxide and water by oxygen-tolerant [NiFe]-hydrogenase during H2 cycling in the presence of O2. J. Am. Chem. Soc. 135, 17897–17905. 10.1021/ja408420d 24180286

[B26] Lea-Smith D. J. Bombelli P. Vasudevan R. Howe C. J. (2016). Photosynthetic, respiratory and extracellular electron transport pathways in cyanobacteria. Biochim. Biophys. Acta Bioenerget. 1857, 247–255. 10.1016/j.bbabio.2015.10.007 26498190

[B27] Livak K. J. Schmittgen T. D. (2001). Analysis of relative gene expression data using real-time quantitative PCR and the 2(-Delta Delta C(T)) Method. Methods 25, 402–408. 10.1006/meth.2001.1262 11846609

[B28] Makowka A. Nichelmann L. Schulze D. Spengler K. Wittmann C. Forchhammer K.. (2020). Glycolytic shunts replenish the calvin–benson–bassham cycle as anaplerotic reactions in cyanobacteria. Mol. Plant. 13, 471–482. 10.1016/j.molp.2020.02.002 32044444

[B29] McIntosh C. L. Germer F. Schulz R. Appel J. Jones A. K. (2011). The [NiFe]-hydrogenase of the cyanobacterium Synechocystis sp. PCC 6803 works bidirectionally with a bias to H2 production. J. Am. Chem. Soc. 133, 11308–11319. 10.1021/ja203376y 21675712

[B30] Messineo L (1966). Modification of the Sakaguchi reaction: Spectrophotometric determination of arginine in proteins without previous hydrolysis. Arch. Biochem. Biophys. 117, 534–540. 10.1016/0003-9861(66)90094-4

[B31] Mullineaux C. W (2014). Co-existence of photosynthetic and respiratory activities in cyanobacterial thylakoid membranes. Biochim. Biophys. Acta Bioenerget. 1837, 503–511. 10.1016/j.bbabio.2013.11.017 24316145

[B32] Nielsen M. Revsbech N. P. Kühl M. (2015). Microsensor measurements of hydrogen gas dynamics in cyanobacterial microbial mats. Front. Microbiol. 6, 726. 10.3389/fmicb.2015.00726 PMC4508582 26257714

[B33] Pandelia M.-E. Ogata H. Lubitz W. (2010). Intermediates in the catalytic cycle of [NiFe] hydrogenase: functional spectroscopy of the active site. Chemphyschem 11, 1127–1140. 10.1002/cphc.200900950 20301175

[B34] Schriek S. Kahmann U. Staiger D. Pistorius E. K. Michel K.-P. (2009). Detection of an L-amino acid dehydrogenase activity in Synechocystis sp. PCC 6803. J. Exp. Bot. 60, 1035–1046. 10.1093/jxb/ern352 19213808 PMC2652061

[B35] Schriek S. Rückert C. Staiger D. Pistorius E. K. Michel K.-P. (2007). Bioinformatic evaluation of L-arginine catabolic pathways in 24 cyanobacteria and transcriptional analysis of genes encoding enzymes of L-arginine catabolism in the cyanobacterium *Synechocystis* sp. PCC 6803. BMC Genom. 8, 437. 10.1186/1471-2164-8-437 PMC2242806 18045455

[B36] Schuller J. M. Birrell J. A. Tanaka H. Konuma T. Wulfhorst H. Cox N.. (2019). Structural adaptations of photosynthetic complex I enable ferredoxin-dependent electron transfer. Science 363, 257–260. 10.1126/science.aau3613 30573545

[B37] SÈtif P. Shimakawa G. Krieger-Liszkay A. Miyake C. (2020). Identification of the electron donor to flavodiiron proteins in Synechocystis sp. PCC 6803 by *in vivo* spectroscopy. Biochim. Biophys. Acta Bioenerget. 1861:148256. 10.1016/j.bbabio.2020.148256 32622739

[B38] Shimakawa G. Shaku K. Nishi A. Hayashi R. Yamamoto H. Sakamoto K.. (2014). Flavodiiron 2 and 4 proteins mediate an oxygen-dependent alternative electron flow in *Synechocystis* sp. PCC 6803 under CO2-limited conditions. Plant Physiol. 167, 472–480. 10.1104/pp.114.249987 25540330 PMC4326736

[B39] Stephan D. P. Ruppel H. G. Pistorius E. K. (2000). Interrelation between cyanophycin synthesis, l-arginine catabolism and photosynthesis in the cyanobacterium *Synechocystis* Sp. strain PCC 6803. Zeitschrift Naturforschung 55, 927–942. 10.1515/znc-2000-11-1214 11204198

[B40] Teeling H. Fuchs B. M. Becher D. Klockow C. Gardebrecht A. Bennke C. M.. (2012). Substrate-controlled succession of marine bacterioplankton populations induced by a phytoplankton bloom. Science 336, 608–611. 10.1126/science.1218344 22556258

[B41] Theune M. L. Hildebrandt S. Steffen-Heins A. Bilger W. Gutekunst K. Appel J. (2021). *In-vivo* quantification of electron flow through photosystem I – Cyclic electron transport makes up about 35% in a cyanobacterium. Biochim. Biophys. Acta Bioenerget. 1862, 148353. 10.1016/j.bbabio.2020.148353 33346012

[B42] Trautmann D. Voss B. Wilde A. Al-Babili S. Hess W. R. (2012). Microevolution in cyanobacteria: re-sequencing a motile substrain of *Synechocystis* sp. PCC 6803. DNA Res. 19, 435–448. 10.1093/dnares/dss024 23069868 PMC3514855

[B43] van Kooten O. Snel J. F. H. (1990). The use of chlorophyll fluorescence nomenclature in plant stress physiology. Photosyn. Res. 25, 147–150. 10.1007/BF00033156 24420345

[B44] Vignais P. M. Billoud B. (2007). Occurrence, classification, and biological function of hydrogenases: an overview. Chem. Rev. 107, 4206–4272. 10.1021/cr050196r 17927159

[B45] Wang Y. Chen X. Spengler K. Terberger K. Boehm M. Appel J.. (2022). Pyruvate:ferredoxin oxidoreductase and low abundant ferredoxins support aerobic photomixotrophic growth in cyanobacteria. Elife 11, e71339. 10.7554/eLife.71339 35138247 PMC8887894

[B46] Wetz M. S. Wheeler P. A. (2003). Production and partitioning of organic matter during simulated phytoplankton blooms. Limnol. Oceanogr. 48, 1808–1817. 10.4319/lo.2003.48.5.1808

[B47] Wetz M. S. Wheeler P. A. (2004). Response of bacteria to simulated upwelling phytoplankton blooms. Mar. Ecol. Prog. Ser. 272, 49–57. 10.3354/meps272049

[B48] Williams J. G. K (1988). “Construction of specific mutations in photosystem II photosynthetic reaction center by genetic engineering methods in Synechocystis 6803,” in Methods in Enzymology (Academic Press), 167, 766–778. 10.1016/0076-6879(88)67088-1

