# Supplementary material for: Synechocystis sp. PCC 6803 Requires the Bidirectional Hydrogenase to Metabolize Glucose and Arginine Under Oxic Conditions
Source: Front Microbiol. 2022 May 31;13:896190. doi: 10.3389/fmicb.2022.896190 (PMC9195167; doi:10.3389/fmicb.2022.896190)
Supplement: Supplementary file 1 [file Data_Sheet_1.PDF]

Supplementary material

Supplementary Table 1: List of strains that were constructed and utilized in this study.

Strain	Marker of genotype	Reference
WT		Trautmann et al. 2012
ΔhoxH	*sll1226:km^R*	Appel et al. 2000
Δhox (ΔHoxEFUYH)	*sll1220, sll1221,sll1222,sll1223,sll1224, ssl2420, sll1225, sll1226::km^R*	This study
ΔhoxW	*slr1876::sp^R*	Hoffmann et al. 2006
Δhox/hoxYH	*sll1220, sll1221,sll1222,sll1223,sll1224, ssl2420, sll1225, sll1226::km^R/ sll1224, sll1226::gm^R*	This study
ΔcydΔcox	*slr1379, slr1380::sp^R, slr1137::em^R*	This study
Δflv3Δflv24ΔcydΔcox	*sll0550::cm^R, sll0219, sll0217::gmR; slr1379, slr1380::sp^R, slr1137::em^R*	This study
Δflv3Δflv24ΔcydΔcoxΔhox	*sll0550::cm^R, sll0219, sll0217::gm^R; slr1379, slr1380::sp^R, slr1137::em^R, sll1220, sll1221,sll1222,sll1223,sll1224, ssl2420, sll1225, sll1226::km^R*	This study
ΔcydΔcoxΔarto	*slr1379, slr1380::sp^R, slr1137::em^R, slr2082, slr2083 :gm^R*	This study
Δndh1 (ΔndhD1ΔndhD2)	*slr0331::km^R, slr1291::cm^R*	Wang et al. 2022
Δsdh (Δsdh1Δsdh2)	*sll1625::gm^R, sll0823::gm^R*	This study
Δndh2 (Δ*ndbA*Δ*ndbB*Δ*ndbC)*	*slr0851::gm^R, slr1743::cm^R, and sll1484::sp^R*	This study

Suppelemtary table 2: List of primers that were utilized in this study.

Primer name	Sequence	Construct
hoxout1	CGTTGTAAAACGACGGCCAGTGCCATTATCTGCCAGTGAAGCCCTT	Construction of Δ*hox*
hoxin1Km	CTTTCTGGCTGGATGATGGGGCGATGATAAAAGATGATTGGGAGAGCCTA	
hoxin2Km	ATCAGAGATTTTGAGACACAACGTGGGGCATCACCGAGGGCATATCT	
hoxout2	GGAAACAGCTATGACCATGATTACGGTTCAGCCAGCAACTAGCCCTTT	
ARTOout1	CTATAGGGCGAATTGGGTACCTGGATCAGCTAATTACCCTAATTAGTA	Constuction of Δarto
ARTOin1Gm	GGTTCGTGCCTTCATCCGTCGACAGCGGGAAAGGGCAGTGCTTGTTT	
ARTOin2Gm	CGCCACCTAACAATTCGGTCGACCCAGCGAATTAAATCTTTGGCAT	
ARTOout2	AGGGAACAAAAGCTGGAGCTCTAGAATTCCACAGTCATAGGCAA	
ndbAout1	CTATAGGGCGAATTGGGTACAATATTTTCGCCGTTGCTATGAA	Constuction of ΔndbA
ndbAin1	GGTTCGTGCCTTCATCCGTCGACGCATGGTCCTCCAACACCACTTT	
ndbAin2	CGCCACCTAACAATTCGGTCGACTAATATATTTGTCCTGGGGGATTT	
ndbAout2	AGGGAACAAAAGCTGGAGCTAGCTATGGTGGGGTTTACCGAA	
ndbBout1	CTATAGGGCGAATTGGGTACCCACCAAAAGGCGATCGCCACTTA	Construction of Δ*ndbB*
ndbBin1	TCAATAATATCGAATTCCTGCAGCCGTGGTCGAGCGTCCGTCATAAT	
ndbBin2	AGCGAGGTGCCGCCATCAAGCTTAAAAATGAACCTTCCTGAGGGAAA	
ndbBout2	AGGGAACAAAAGCTGGAGCTATGGGGGTGGTAATAGGCCATT	
ndbCout1	CTATAGGGCGAATTGGGTACAATCACCGCCGCCCAGGTTCAAT	Construction of Δ*ndbC*
ndbCin1	TTGGCACCCAGCCTGCGCGAAAAGTGGGGCCAATTTCCTGGAAA	
ndbCin2	ATCCGCATTAAAATCTAGCGAGGGCACCGGAAAGGGAAAGGGCTCCTT	
ndbCout2	AGGGAACAAAAGCTGGAGCTGGACAATGATGGGATGGAGGGTAT	
Flv3out1	TGGTGTTGGCCCATCCAGCCCGGTA	Construction of Δ*flv3*
Flv3in1	AAATGCTTCAATAATATCGAATTCCTGCAGAGGGGGGTAGTGAACATGCCGTACT	
Flv3in2	ATCCGTTAGCGAGGTGCCGCCATCAAGCTTCATCGCAAAGTCGGCAATTATTACT	
Flv3out2	AACCCTGGGGTCAAAACTCTCCGGG	
0217out1	CCACAAAGTTTTCTATGGTTAC	Construction of Δ*flv24*
0217in1	GGTTCGTGCCTTCATCCGTCGACTCTGTATGATTGACGATCAAATA	
0219in1	CGCCACCTAACAATTCGGTCGACCGATCATGTCCTGGTTTATGCAA	
0219out1	CAAAACTGACGTCAGCATGGCATT	
cydout1	CTATAGGGCGAATTGGGTACAGAAGGAGTTTACGATCGCCAA	Construction of Δ*cyd*
cydin1	TTGGCACCCAGCCTGCGCGATTACTCAAAAAATCCTGCATCTGTAA	

cydin2	ATCCGCATTAAAATCTAGCGAGGGCAAAATTGTCACCGACTAGGGAGTT	
cydout2	AGGGAACAAAAGCTGGAGCTTGCAACGGGTCAGCATCCAATTT	
cox1out1	CTATAGGGCGAATTGGGTACAATTACGGTTAAAGCAGGAT	Construction of Δ*cox*
coxin1	AGAGATTTATCTAATTTCTTTTTTCGTCGACGATTCTCAGCGGCAATAGTCATAAA	
coxin2	AATTATTTAATAAGTAAGTCGACGATGCGGCAGGAAGTTAGTTT	
coxout2	AGGGAACAAAAGCTGGAGCTAAACTAACTTCCTGCCGCATC	
sdhB1out1	CTATAGGGCGAATTGGGTACGACAGTTCTGCTTCCGGTCAA	Constuction of the Δ*sdh1*
sdhB1in1	GGTTCGTGCCTTCATCCGTCGACATTTTGCAAACAATTTCCATGGTA	
sdhB1in2	CGCCACCTAACAATTCGGTCGACTTCGTTTATTTGACTTGATGGAT	
sdhB1out2	AGGGAACAAAAGCTGGAGCTTGTGACCTGGCAATTTTGATGT	
sdhB2out1	CTATAGGGCGAATTGGGTACGAGAGTTTGGCCCAAAAGTTGA	Constuction of the Δ*sdh2*
sdhB2in1	TCAATAATATCGAATTCCTGCAGCTGTTGCGGTTTTTGGCGCAA	
sdhB2in2	AGCGAGGTGCCGCCATCAAGCTTGCGGAAAAACTTGCCAATTTCT	
sdhB2out2	AGGGAACAAAAGCTGGAGCTTAACCGTTCCAATCGACTTT	
hoxE_89f	TTTTGGCTACCTGGAAGAGG	primers used for real-time PCR
hoxE_89r	AAAAAGTCGCCACTCCAAAC	
hoxF_84f	TTTTGCCCTCACAGGAAAAG	
hoxF_84r	ATTTCCTCCACCACTTGTCG	
sll1222_84f	CGCTCAACGGAAATGATACC	
sll1222_84r	TATCCCCCACATGGCAAG	
hoxU_93f	CGAAGGCAATCATGTCTGTG	
hoxU_93r	AATCGGCTGTGATCCATACC	
hoxY_92f	CGGCTGTCATATGTCCTTCC	
hoxY_92r	GATCAGAACCAACGGGACTG	
sll1223_100f2	CCAAACGAATCATGGAGAAG	
sll1223_100r2	TGGCCAAAAACTCCTTTG	
HoxH_88f	GGACAGTGATCCCGCTACTC	
HoxH_88r	AAATTGCCGTAACCGAATACC	
16S_91f	AAGTCATCATGCCCCTTACG	
16S_91r	ATGGGATTCGCTTACTCTCG	

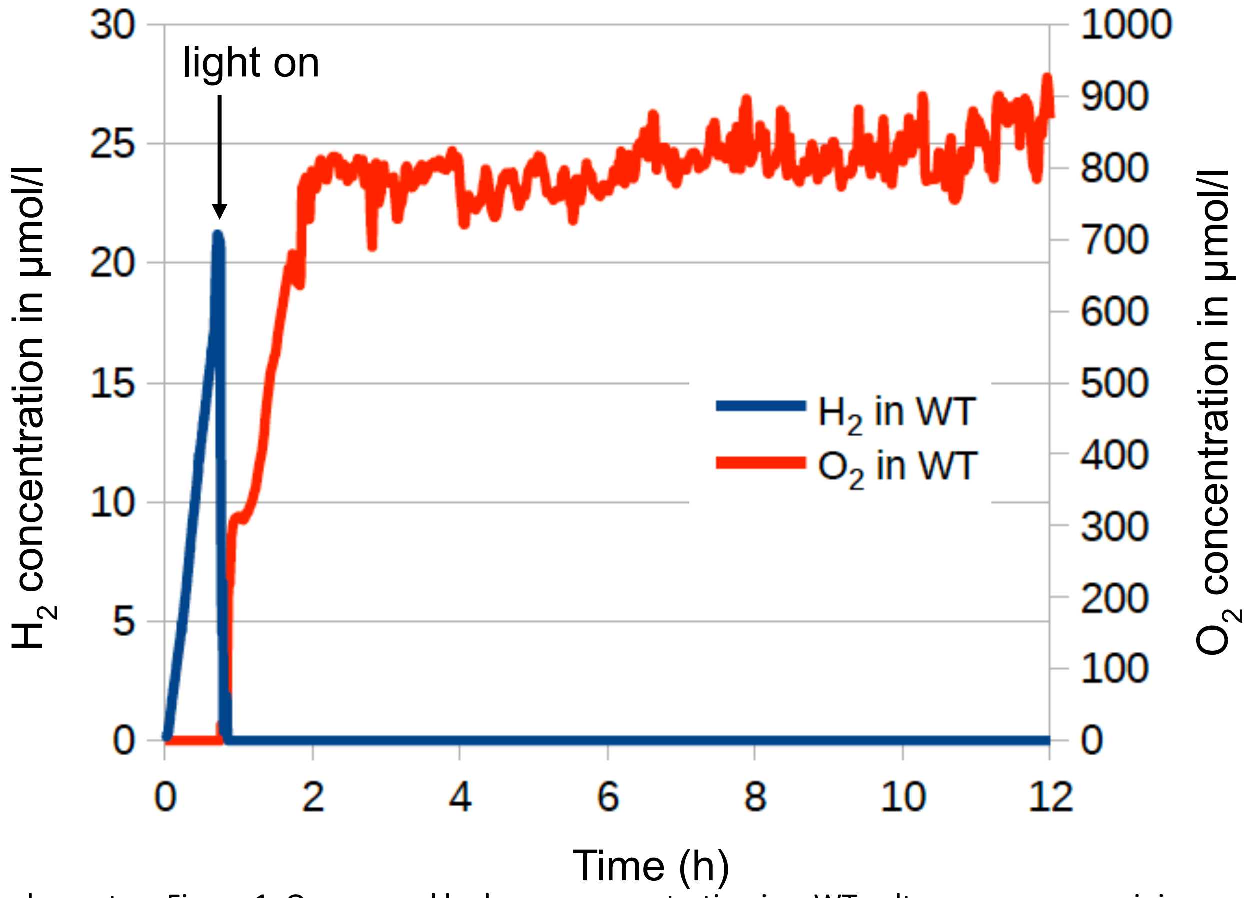

Supplementary Figure 1: Oxygen and hydrogen concentration in a WT culture grown on arginine and glucose without purging, in order to exclude outgasing of hydrogen (for details see text). The cells were brought under dark, anoxic conditions from 0 to 0.73h in order to prove their ability to produce fermentative hydrogen. The hydrogen concentration reached 21 µmol in this period. As soon as the cells were illuminated hydrogen was consumed. The cultures were purged with ambient air until they were saturated with oxygen (0.85h to 0.95h). The aeration was turned off and the cultures were left under continous light. The oxygen concentration was stable and no hydrogen production could be detected under these conditions.

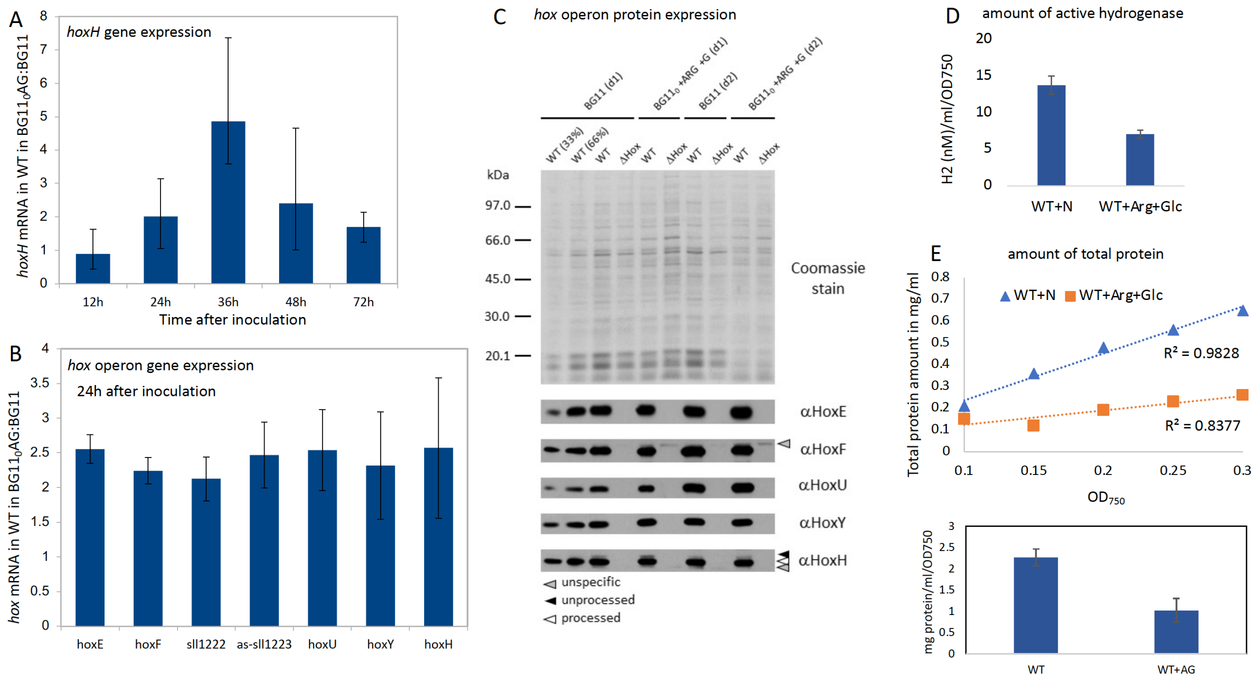

Supplementary Figure 2: Transcription and expression of the hydrogenase subunits in cultures cultivated on arginine and glucose. (A) Amount of *hoxH* mRNA in WT on arginine and glucose ($BG11_0AG$) relative to WT on nitrate (BG11). 16S rRNA was utilized as reference gene. (B) Amount of *hox operon* mRNA in WT on arginine and glucose ($BG11_0AG$) relative to WT on nitrate (BG11). 16S rRNA was utilized as reference gene. (C) Protein expression of the HoxE, F, U, Y, H subunits in the WT on arginine and glucose ($BG11_0AG$) relative to WT on nitrate (BG11). *Δhox*, in which the *hoxEFUYH* operon was deleted, was utilized as a negative control to demonstrate the specificity of the utilized antibodies. (D) Amount of active hydrogenase measured upon the addition of methylviologen in WT on nitrate (N) and on arginine and glucose (AG). Two cultures with equal optical densities (OD_{750}) are compared. (E) Total amount of protein in WT on nitrate (N) and on arginine and glucose (AG) relative to OD_{750}.

In order to quantify the amount of functional hydrogenase, which should correlate with Hox protein levels (Supplementary Figure 2C), hydrogen production was determined upon addition of the artificial electron donor methylviologen (MV). The cells cultivated on arginine and glucose contained only about half the amount of hydrogenase activity compared to cells that were cultivated on nitrate (Supplementary Figure 2D). Obviously, these numbers don´t give a conclusive picture. Even though transcription of the *hox* operon was enhanced in cells that were cultivated on arginine and glucose (Supplementary Figure 2A and 2B), Hox protein levels remained

largely unchanged or declined (Supplementary Figure 2C). For some reasons, the demand for additional hydrogenase expression, which was observed in the increased transcription levels, was not implemented on a protein level and did thus not correlate to increased hydrogenase activity. Whereas transcript levels were related to equal amounts of 16S rRNA as reference gene, protein contents were related to total protein amount and MV based hydrogenase activity measurements to equal optical densities OD_{750} of cultures. As immunoblots and hydrogenase activity measurements, which were both utilized to determine protein levels gave different results, we tested the amount of total protein relative to OD_{750}. Total protein amount relative to OD_{750} was reduced to 44 % in photomixotrophic cultures on arginine and glucose (Supplementary Figure 2E). This explains why hydrogenase activity measurements did not correlate well with immunoblots. It might furthermore indicate that protein biosynthesis might be disturbed, which could explain why additional *hox* transcriptions were obviously not translated. However, this explanation is speculative and requires further testing.

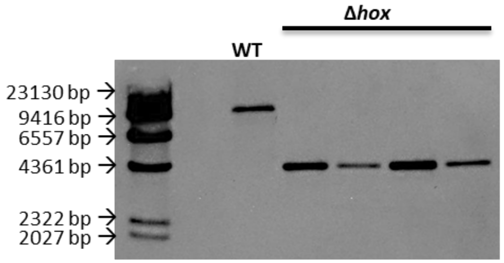

Supplementary Figure 3: Southernblot of the WT and the Δ*hox* strain with a probe against *hox*: Expected band sizes were 11300 bp for the WT and 5200 bp for Δhox.

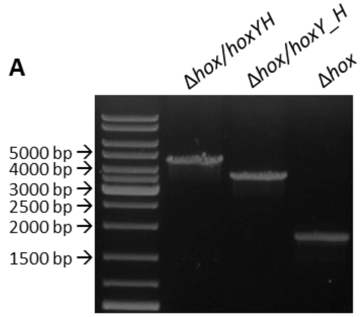

Supplementary Figure 4: PCR to test for segregation of Δ*hox*/*hoxYH*. A probe against *hox* was utilized. The expected band sizes were: Δ*hox*: 1750 bp; Δ*hox*/*hoxYH*: 4400 bp. Strain Δ*hox*/*hoxY_H* is not relevant for this study.

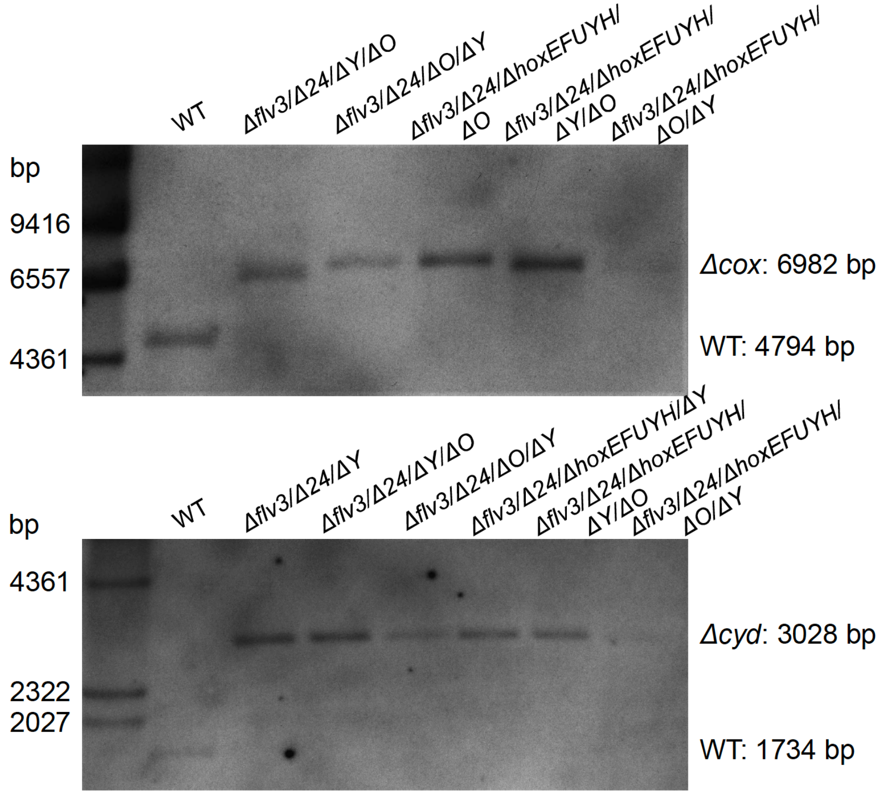

Supplementary Figure 5: Southernblot for testing segregation of the mutants as indicated. Probes against cox and cyd were utilized. Expected band sizes for the probe against cox were 4794 bp for the WT and 4794 bp for Δ*cox*. For the probe against *cyd* 1734 bp were expected for the WT and 3028 bp were expected for the mutant. Cyd is abbreviated as Y and Cox as O. Only the WT and Δ*flv3*Δ*flv24*Δ*cox*Δ*cyd* (here labeled as Δ*flv3*Δ*flv24*Δ*O*Δ*Y)* and Δ*flv3*Δ*flv24*Δ*cox*Δ*cyd*Δ*hox* (here labeled as Δ*flv3*Δ*flv24*Δ*hoxEFUYH*Δ*O*Δ*Y)* are relevant for this study.

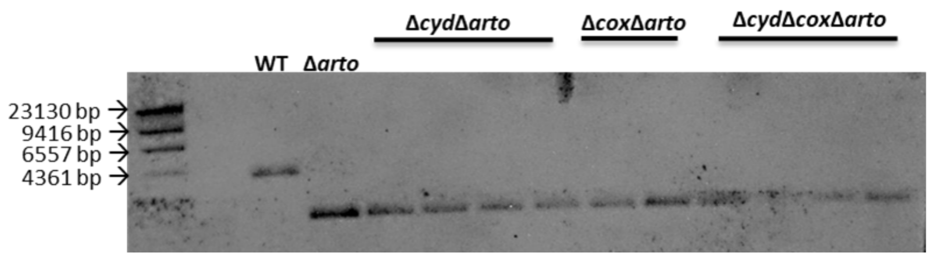

Supplementary Figure 6: Southernblot of the WT and different mutants as indicated with a probe against *arto*: Expected band sizes were 3860 bp for the WT and 1870 bp for Δ*arto*.

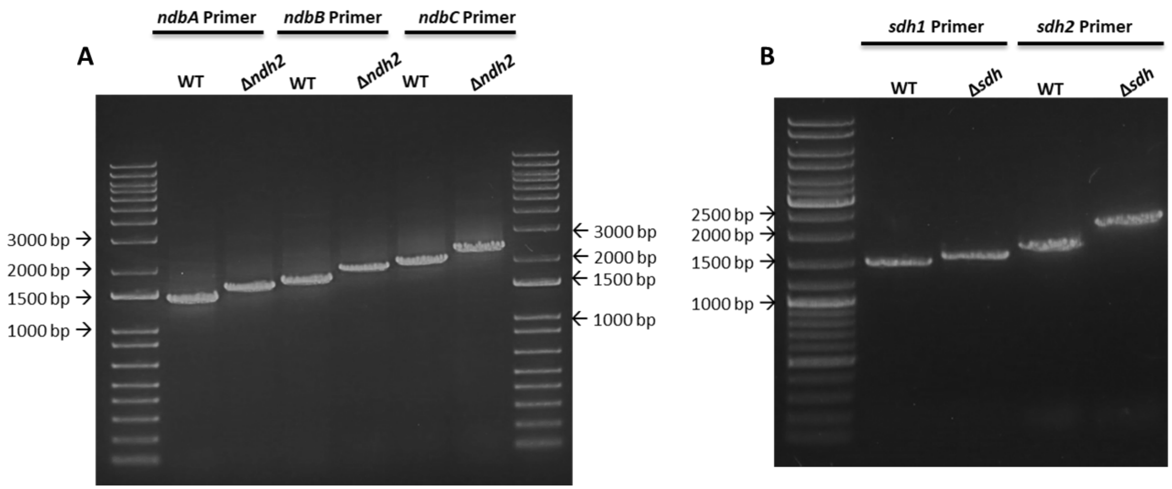

Supplementary Figure 7: PCR with genomic DNA of different mutants in order to test for their segregation. (A) Segregation of Δ*ndh2* (Δ*ndbA*Δ*ndbB*Δ*ndbC)* and (B) segregation of Δ*sdh* (Δ*sdh1*Δ*sdh2)*. Expected sizes of PCR products: *ndbA* primer pair WT: 1388 bp, Δ*ndh2*: 1550 bp; *ndbB* primer pair; WT: 1663 bp, Δ*ndh2*: 1884 bp; *ndbC* primer pair; WT: 2000 bp, Δ*ndh2*: 2270 bp. The expected band sizes for Δ*sdh* were: *sdh1* primer pair; WT: 1470 bp, Δ*sdh*: 1530 bp; *sdh2* primer pair; WT: 1680 bp; Δ*sdh*: 2390 bp. Δ*ndh2* and Δ*sdh* were completely segregated.

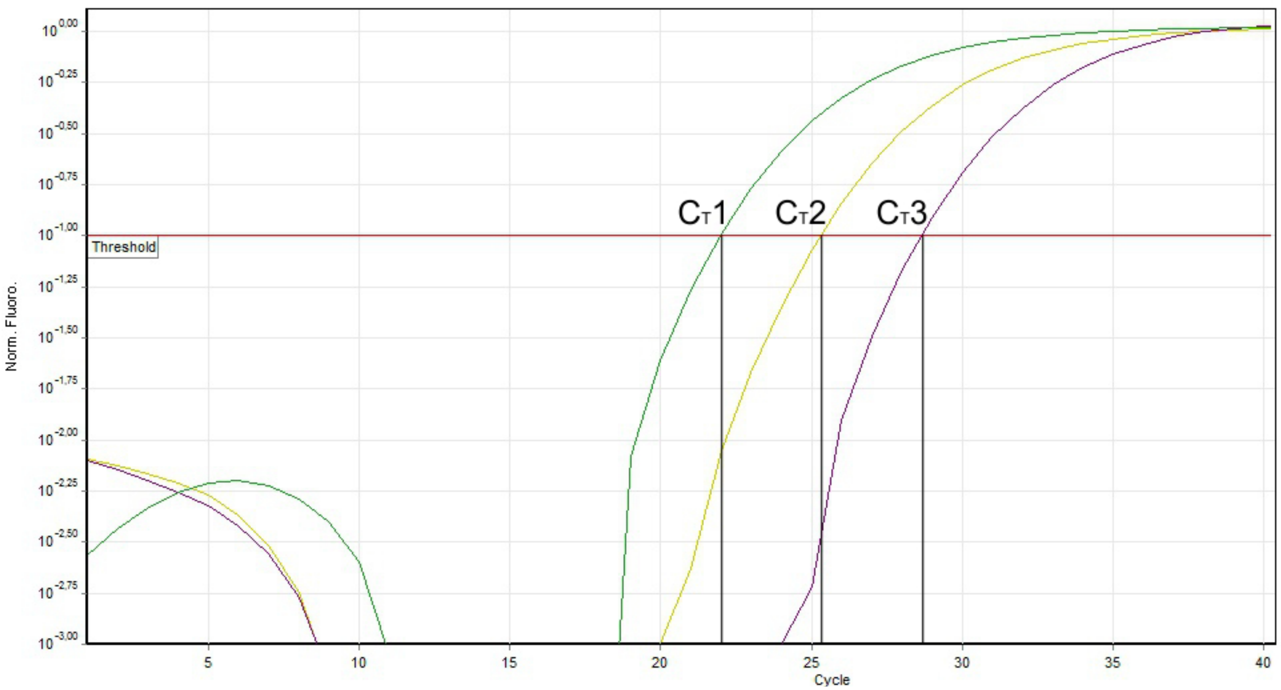

Supplementary Figure 8: CT values were determined for each sample. The graph shows a representative amplification plot for three DNA dilutions (1: 1:30, 2: 1:300; and 3: 1:3000). The threshold was set to a normalized fluorescence of 10^{-1} for all samples.
